# Assessing the role of lipid-lowering therapy on multi-cancer prevention: A mendelian randomization study

**DOI:** 10.3389/fphar.2023.1109580

**Published:** 2023-04-19

**Authors:** Yu Min, Xiaoyuan Wei, Zheran Liu, Zhigong Wei, Yiyan Pei, Ruidan Li, Jing Jin, Yongllin Su, Xiaolin Hu, Xingchen Peng

**Affiliations:** ^1^ Department of Biotherapy and National Clinical Research Center for Geriatrics, Cancer Center, West China Hospital, Sichuan University, Sichuan, China; ^2^ Cancer Center, and State Key Laboratory of Biotherapy, Department of Head and Neck Oncology, Department of Radiation Oncology, West China Hospital, Sichuan University, Sichuan, China; ^3^ Department of Rehabilitation, Cancer Center, West China Hospital, Sichuan University, Sichuan, China; ^4^ West China School of Nursing, West China Hospital, Sichuan University, Sichuan, China

**Keywords:** statin, causality, cancer, GWAS, mendelian randomization

## Abstract

**Background**: Statin use for cancer prevention has raised wide attention but the conclusions are still controversial. Whether statins use have exact causal effects on cancer prevention remains unclear.

**Methods**: Based on the Genome-Wide Association Studies (GWAS) datasets from the large prospective UK Biobank and other consortium databases, two-sample mendelian randomization (MR) analysis was conducted to explore the causal effects of statins use on varied site-specific cancer risks. Five MR methods were applied to investigate the causality. The stability, heterogeneity, and pleiotropy of MR results were also evaluated.

**Results**: The atorvastatin use could increase the risk of colorectal cancer (odd ratio (OR) = 1.041, *p* = 0.035 by fixed-effects inverse variance weighted (IVW) method (IVW_FE_), OR = 1.086, *p* = 0.005 by weighted median; OR = 1.101, *p* = 0.048 by weighted mode, respectively). According to the weighted median and weighted mode, atorvastatin could modestly decrease the risk of liver cell cancer (OR = 0.989, *p* = 0.049, and OR = 0.984, *p* = 0.004, respectively) and head and neck cancer (OR = 0.972, *p* = 0.020). Besides, rosuvastatin use could reduce the bile duct cancer risk by 5.2% *via* IVW_EF_ method (OR = 0.948, *p* = 0.031). No significant causality was determined in simvastatin use and pan-cancers *via* the IVW_FE_ or multiplicative random-effects IVW (IVW_MRE_) method if applicable (*p* > 0.05). There was no horizontal pleiotropy observed in the MR analysis and the leave-one-out analysis proved the stability of the results.

**Conclusion**: The causalities between statin use and cancer risk were only observed in colorectal cancer and bile duct cancer in the European ancestry population. Future works are warranted to provide more robust evidence for supporting statin repurposing for cancer prevention.

## Introduction

In past decades, general cancer-associated morbidity and mortality have grown rapidly and become a major burden for public health management (Sung et al., 2021; Siegel et al., 2022). Thus, early prevention, screening, and diagnosis become the effective strategies for reducing the cancer burden on the population (Rebbeck et al., 2018). As for cancer prevention, especially in terms of chemoprevention, drugs that were frequently prescribed for metabolic and cardiovascular diseases were noticed to have positive effects on the anticancer process (Gronich and Rennert, 2013; Morales and Morris, 2015).

**FIGURE 1:**
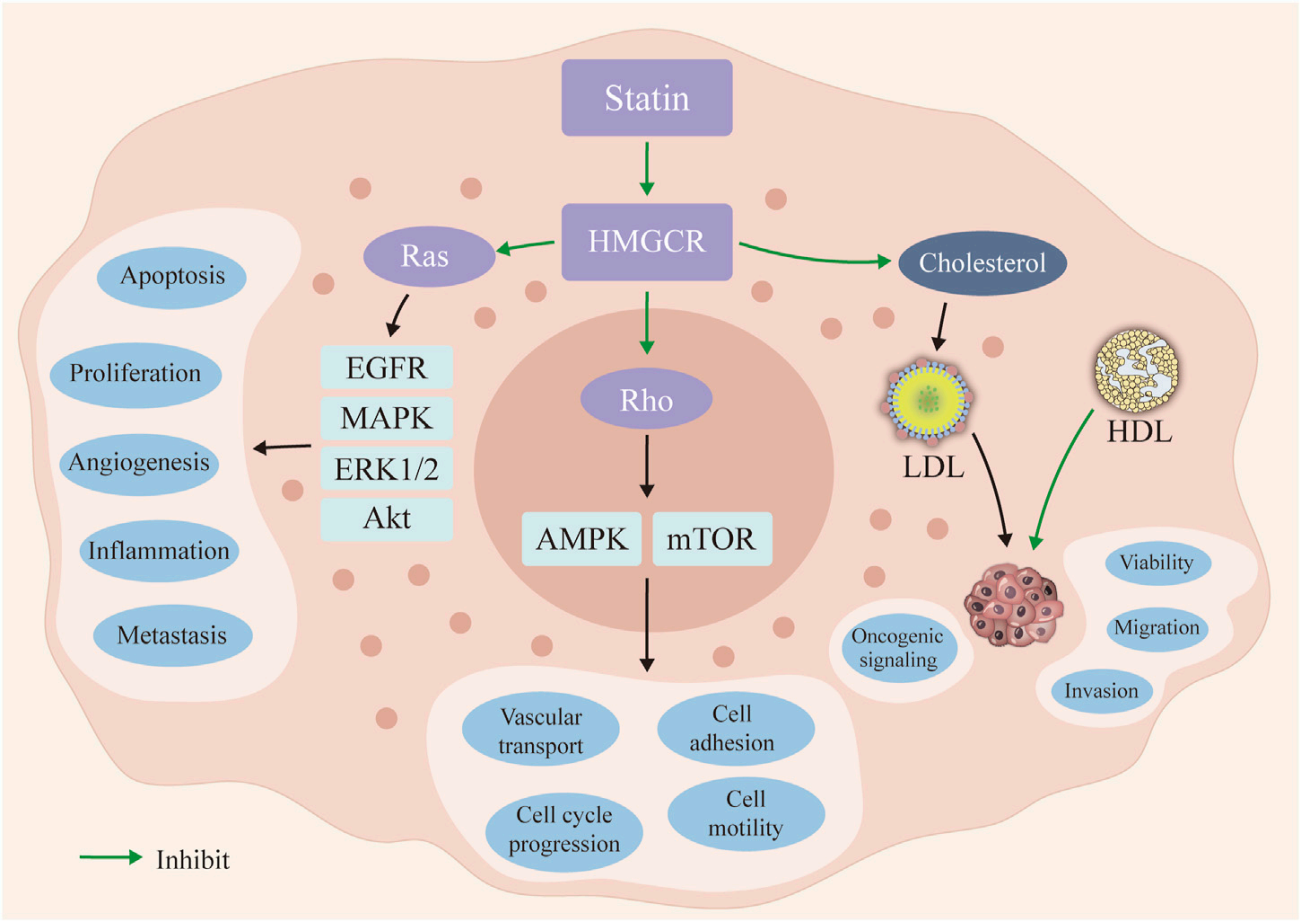
The potential mechanisms of statin use in cancer prevention. Statin could inhibit the 3-hydroxy-3-methyl-glutaryl-CoA (HMG-CoA) reductase (HMGCR) and further inhibit the Ras and Rho signaling pathways. Then, the occurrence and progression of the cancer cells could be inhibited in statin use. The reduced serum level of LDL inhibits the oncogenic signaling activation. LDL: low density lipoprotein; HDL: high density lipoprotein.

Statins, the inhibitors of 3-hydroxy-3-methyl-glutaryl-CoA (HMG-CoA) reductase (HMGCR), are commonly used as lipid-lowering drugs for atherosclerosis (Ziaeian and Fonarow, 2017). Recently, statins have also received increasing attention owing to the rate-limiting enzyme in the mevalonate pathway (a pathway controlling a range of cell signaling molecules with the potential to regulate carcinogenesis) for cancer prevention (Saka-Herrán et al., 2022; Khazaaleh et al., 2022; Zhang et al., 2019; Longo et al., 2022; Cheung et al., 2023) (Figure 1). Detailly, preclinical research revealed that statins might inhibit the cancer cell proliferation *via* countering the cell cycle at the G1-S phase and enhancing the cell apoptosis (Ahmadi et al., 2017). Meanwhile, statins could also inhibit Ras/Rho pathways to further inhibit the multiple carcinogenic signaling pathways (Ahmadi et al., 2017; Patel and Kashfi, 2022). Clinically, although numerous population-based studies have described the anticancer properties of statins, the evidence for the anticancer effects of statins is still debatable. Notably, results from one large-scale European population-based study (3,118 biliary tract cancers cases and 15,519 controls), Liu et al. demonstrated that statins use could decrease by nearly 12% (odd ratio (OR) = 0.88), 95% confidence interval (CI): 0.79–0.98) risk of biliary tract cancer (Liu et al., 2019). Similarly, evidence from the Asian population, Chiu et al. determined a protective role of statin use for liver cancer prevention, statins use could significantly reduce the liver cancer risk by 38% (OR = 0.62, 95% CI = 0.42–0.91), compared with no use of statins (Chiu et al., 2011). Conversely, no association between statin use and cancer prevention effects was noticed in recent studies (Baandrup et al., 2015; Wang et al., 2019; Saka-Herrán et al., 2022). Specifically, with a relatively smaller sample size, Chan et al. did not find any beneficial role of statin use on esophageal cancer prevention (Chan et al., 2013). Moreover, evidence from one recent prospective study, the beneficial role of statin use on skin cancer risk was still questionable (Al Rahmoun et al., 2022). More importantly, some researchers even highlighted the potential risk of long-term statins use in developing invasive ductal carcinoma (OR = 1.83) and invasive lobular carcinoma (OR = 1.97) (McDougall et al., 2013). Thus, the disparity results forward us to find a more comprehensive analysis for evaluating the role of statin use in cancers (Ahmadi et al., 2017; Patel and Kashfi, 2022).

Notably, the application of Mendelian randomization (MR) analysis (Sekula et al., 2016; Hemani et al., 2018) based on Genome-wide association studies (GWAS) data was widely used in different fields of public health, which could also help us to investigate the causality by correlating GWAS data of statins use and cancers at the genetic level.

Currently, there are scarce reports in the literature regarding the possible role of statin use in cancer risk based on MR methods. Herein, we aim to evaluate the causal effect of three statin types on multiple cancer risks *via* the two-sample MR analysis. The results could provide more robust evidence for making clinical decisions.

## Materials and methods

### Data source

In this study, statin use is investigated *via* three specific prescribed drugs including atorvastatin, simvastatin, and rosuvastatin, which represent the different lipid solubility of statin. The GWAS datasets for statin use in the present study are all derived from the Medical Research Council-Integrative Epidemiology Unit (MRC-IEU) GWAS database (https://gwas.mrcieu.ac.uk/). (Hemani et al., 2018). The GWAS data of atorvastatin (ID: ukb-b-10008), simvastatin (ID: ukb-b-11268), and rosuvastatin (ID: ukb-b-13664), therefore, downloaded from the “MR-base”, a platform for MR (Hemani et al., 2018).

Meanwhile, the GWAS data of cancers is also extracted from the “MR-base”. To reduce the bias of the GWAS data analysis, cancer types that satisfied the following three criteria were included: 1) it is available in the “MR-base”; 2) the GWAS datasets of the cancer were up to date, within 5 years at least; 3) the number of SNPs was over one million. Therefore, a total of 13 types of cancers, including bladder cancer (ieu-b-4874), lung cancer (ieu-b-4954), bile duct cancer (ieu-b-4915), liver cell cancer (ieu-b-4953), cervical cancer (ieu-b-4876), colorectal cancer (ieu-b-4965), ovarian cancer (ieu-b-4963), non-melanoma skin cancer (ieu-b-4959), melanoma (ieu-b-4969), prostate cancer (ieu-b-85), breast cancer (ieu-a-1130), esophagus cancer (ieu-b-4960), and head and neck cancer (ieu-b-4912) were derived from the UK Biobank Consortium, Breast Cancer Association Consortium (BCAC), the Prostate Cancer Association Group to Investigate Cancer Associated Alterations in the Genome (PRACTICAL) Consortium, and the “MR-base” with unknown Consortium. The detailed information on the GWAS datasets were summarized in Table 1. Moreover, we defined atorvastatin, simvastatin, and rosuvastatin were the exposure and different cancers were the outcome to explore the causal effects of these two events.

**TABLE 1:** The list of Genome-wide summary association studies (GWAS) included in the Mendelian randomization (MR) study.

Subtype	GWAS ID	Years	Sex	Sample size	nCases	nControl	No. SNPs	Consortium
**Statin**								
Atorvastatin	ukb-b-10008	2018	M/F	462,933	13,851	449,082	9,851,867	MRC-IEU
Simvastatin	ukb-b-11268	2018	M/F	462,933	52,427	410,506	9,851,867	MRC-IEU
Rosuvastatin	ukb-b-13664	2018	M/F	462,933	2,870	460,063	9,851,867	MRC-IEU
**Cancer site**								
Bladder	ieu-b-4874	2021	M/F	373,295	1,279	372,016	9,904,926	NA
Lung	ieu-b-4954	2021	M/F	374,687	2,671	372,016	11,078,115	UK Biobank
Bile duct	ieu-b-4915	2021	M/F	372,366	350	372,016	7,687,713	UK Biobank
Liver cell	ieu-b-4953	2021	M/F	372,184	168	372,016	6,304,034	UK Biobank
Cervical	ieu-b-4876	2021	F	199,086	563	198,523	8,506,261	NA
Colorectal	ieu-b-4965	2021	M/F	377,673	5,657	372,016	11,738,639	UK Biobank
Ovarian	ieu-b-4963	2021	F	199,741	1,218	198,523	9,822,229	UK Biobank
Non-melanoma	ieu-b-4959	2021	M/F	395,710	23,694	372,016	12,321,875	UK Biobank
Melanoma	ieu-b-4969	2021	M/F	375,767	3,751	372,016	11,396,019	UK Biobank
Prostate	ieu-b-85	2018	M	140,254	79,148	61,106	20,346,368	PRACTICAL
Breast	ieu-a-1130	2017	F	89,677	46,785	42,892	10,680,257	BCAC
Oesophagus	ieu-b-4960	2021	M/F	372,756	740	372,016	8,970,465	UK Biobank
Head and neck	ieu-b-4912	2021	M/F	373,122	1,106	372,016	9,655,080	UK Biobank

Abbreviation: F/M: female and male; SNPs: single-nucleotide polymorphisms.

### SNPs selection and assumption

**FIGURE 2:**
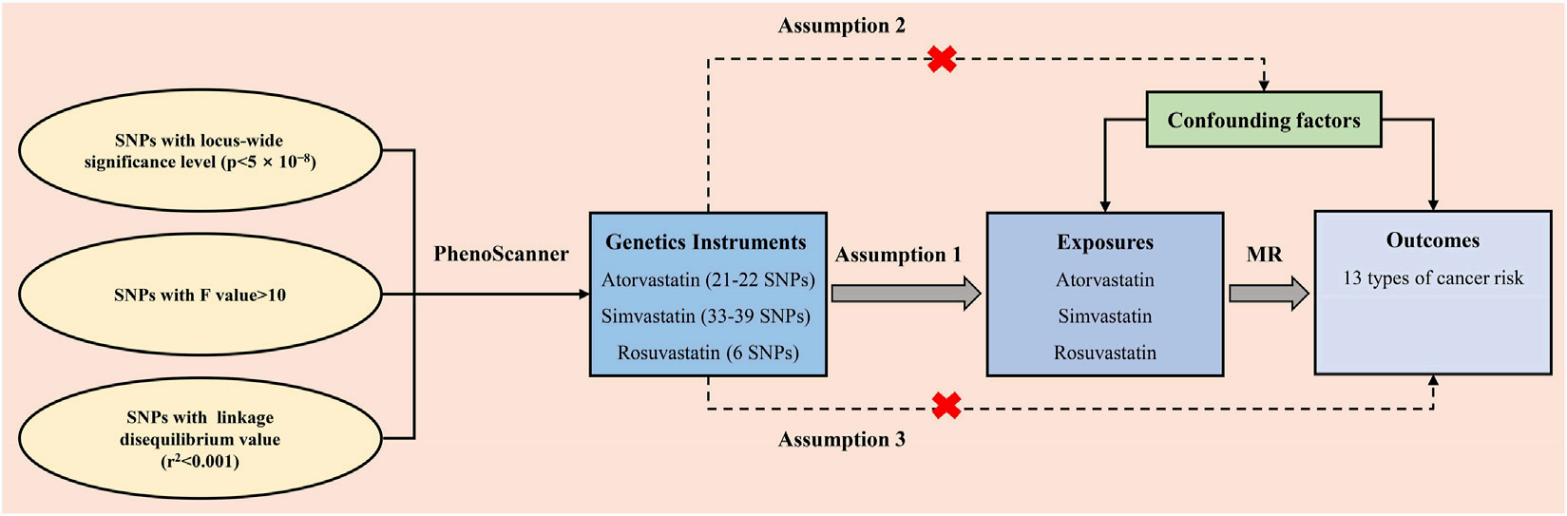
The three assumptions for the two-sample Mendelian randomization analysis in this study. SNP: single-nucleotide polymorphism; MR: Mendelian randomization.

Our study satisfied three assumptions of MR analysis (Figure 2) (Davey Smith and Hemani, 2014). First and foremost, the SNPs selected for MR analysis must be strongly associated with the exposure. To ensure satisfy the assumption, only SNPs whose *p*-values were below the locus-wide significance level (5 × 10^−8^) were included for analysis. To ensure the robust association between instrumental variables and exposure factors, we excluded instrumental variables with F values [formula: (R2/(R2-1)] *[(N-K-1)/K)] <10. Second, the chosen instrumental variables must undergo the independence test. The SNP linkage disequilibrium value (*r* ^2^) was set to 0.001 and the genetic distance was 10,000 kb to eliminate the linkage disequilibrium impact and keep the independence of selected instrument variables. Besides, Each SNP was screened at “PhenoScanner” (Staley et al., 2016), a publicly available database of human genotype-phenotype associations, and Genome-wide SNPs significantly associated with the other diseases and outcomes were eliminated (http://www.phenoscanner.medschl.cam.ac.uk/). The mentioned instrumental variables selection guarantees the quality of our study.

### Two-sample mendelian randomization analysis

To date, numerous statistical methods are available for conducting the two-sample MR analysis. In the present study, five frequently used methods including MR-Egger, Inverse variance weighting (IVW, including Fixed-effect: IVW_FE_ and multiplicative random-effects: IVW_MRE_), weighted median, and weighted mode were enrolled to evaluate the causal effects between statin and pan-cancer risk. As the fundamental analysis method, IVW is a time-honored method for combining the Wald ratio estimates of all relevant instrumental variables. This strategy is analogous to using weighted linear regression to probe the ties between the instrumental factors and the result. The intercept of the instrumental variables is constrained to zero. IVW can obtain unbiased estimates of the status without horizontal pleiotropy. Under the premise of Instrument Strength Independent of Direct Effect (Burgess and Thompson, 2017), the MR-Egger method can primarily demonstrate the dosage relationship between instrumental variables and outcomes while accounting for some pleiotropy. The class one error rate can be lowered using the weighted median method, which also permits the possibility of invalidity for some specific genetic variants. Even if certain instrumental variables did not satisfy the requirements of the MR technique for causal inference, the weighted mode approach remains valid when the vast majority of instrumental variables with identical causal estimates are valid. If the results of these methods are inconsistent, IVW is given priority to be the main findings. Besides, in the rosuvastatin group, there was no satisfied SNP determined in breast and prostate cancers. Thus, the MR analysis was not performed for these two cancer types in the rosuvastatin group.

### Heterogeneity, pleiotropy, and sensitive analyses

Furthermore, pleiotropy (refers to a genetic variant with more than one independent phenotypic effect), which might affect the causal effects, was evaluated by the method of MR Egger. To verify the conformity of each SNP, the heterogeneity test was performed through MR Egger and IVW methods to calculate Cochran Q statistics and find the heterogeneity among genetic variants (Bowden et al., 2015). If the heterogeneity was statistically significant (*p* < 0.05), IVW_MRE_ method is applied to perform the analysis. The Leave-one-out analysis was further performed by reducing the genetic variants one by one, and MR analysis was performed by the rest. The causal relationship would be credible and stable if the result of the leave-one-out analysis conformed to that of the global IVW analysis. It was determined that a causal effect was nominal when the *p*-value was between 0.05 and the corrected value.

### Statistical analysis

The two-tailed *p*-value <0.05 was considered statistically significant. All of the statistical analyses were performed by R (version 4.1.2, https://www.rproject.org/) *via* the “TwoSampleMR” package (the package could be downloaded from the website: https://github.com/MRCIEU/TwoSampleMR).

## Results

### The selected SNPs in MR analysis

Based on the European population, there were 9,851,867 SNPs detected in three different statin use groups. After selection, there were 23 instrumental variables in the atorvastatin group, 41 instrumental variables in the simvastatin group, and 6 instrumental variables in the rosuvastatin group, which satisfied the locus-wide significance level (*p* < 5*10^–8^), used to perform the two-sample MR analysis. Among three exposure datasets, there were 21–22 SNPs calculated for different cancer outcomes in the atorvastatin group (Supplementary Table S1), 33–39 SNPs calculated for different cancer outcomes in the simvastatin group (Supplementary Table S2), and 6 SNPs calculated for different cancer outcomes in simvastatin group (Supplementary Table S3), respectively. Each SNP singly estimated the causal effect of statin use on pan-cancer risk by using the Wald ratio method, which was represented in the forest plot (Supplementary Figures S1-S3).

### Primary two-sample MR analysis

According to the IVW_FE_ analysis, atorvastatin use had a slightly causal effect on liver cell cancer risk (OR = 0.993, 95% CI: 0.986–0.999, *p* = 0.029). Similarly, each standard deviation increase in genetically determined atorvastatin use could slightly decrease the risk of liver cell cancer (OR = 0.989, 95% CI: 0.978–0.999, *p* = 0.049, and OR = 0.984, 95% CI: 0.970–0.998, *p* = 0.004, respectively) and head and neck cancer (OR = 0.972, 95% CI: 0.949–0.995, *p* = 0.020) by weighted median and weighted mode methods (Supplementary Table S4). By contrast, atorvastatin use could increase the risk of colorectal cancer (OR = 1.041, *p* = 0.035 by IVW_FE_ method, OR = 1.086, *p* = 0.005 by weighted median; OR = 1.101, *p* = 0.048 by weighted mode, respectively) (Supplementary Table S4). No significant causal effect was determined in atorvastatin use on other cancer risks (*p* > 0.05), while a difference that nearly reached statistical significance was observed in the outcome for head and neck cancer (odd ratio (OR) = 0.983, 95% confidence interval (CI): 0.967–1.000, *p* = 0.052) (Table 2).

**TABLE 2:** Two sample MR analysis results of atorvastatin use and pan-cancer *via* Fixed-effect inverse variance weighted (IVW_EF_) method.

Outcome	No. SNPs	β	SE_β_	OR (95% CI)	*p*
Bladder	22	−0.004	0.009	0.996 (0.978-1.014)	0.679
Lung	22	−0.012	0.013	0.988 (0.963-1.014)	0.369
Bile duct	21	−0.009	0.005	0.991 (0.982-1.001)	0.072
Liver cell	21	−0.007	0.003	0.993 (0.986-0.999)	**0.029**
Cervical	22	−0.012	0.012	0.988 (0.966-1.011)	0.306
Colorectal	22	0.040	0.019	1.041 (1.003-1.081)	**0.035**
Ovarian	22	0.005	0.017	1.005 (0.972-1.039)	0.758
Non-melanoma	22	−0.035	0.037	0.966 (0.899-1.038)	0.344
Melanoma	22	0.009	0.016	1.009 (0.978-1.041)	0.573
Prostate	22	0.878	0.568	2.405 (0.791-7.316)	0.122
Breast	21	0.372	0.719	1.451 (0.354-5.944)	0.605
Oesophagus	22	0.003	0.007	1.003 (0.989-1.017)	0.700
Head and neck	22	−0.017	0.009	0.983 (0.967-1.000)	0.052

**Abbreviation**: MR: mendelian randomization; SNPs: single-nucleotide polymorphisms; SE: standard error; OR: odd ratio, CI: confidence interval.

Bold values indicate statistical significance (*p* < 0.05).

Notably, simvastatin use showed a significant causal effect on prostate cancer risk (OR = 1.714, 95% CI: 1.085–2.708, *p* = 0.021) *via* the IVW_FE_ method (Table 3). No significant causality was determined in simvastatin use and cancer risk by other supplementary MR analyses (Supplementary Table S5). Regarding the causality among rosuvastatin use and cancer risk, the bile duct cancer risk could be decreased by 5.2% *via* IVW_EF_ method (OR = 0.948, 95% CI: 0.903–0.995, *p* = 0.031), and the liver cancer risk could be reduced by 4.7% *via* IVW_EF_ method (OR = 0.953, 95% CI: 0.921–0.985, *p* = 0.05) (Table 4), by 6.2% *via* the weighted median method (OR = 0.938, 95% CI: 0.897–0.981, *p* = 0.005) and by 7.3% *via* the weighted model method (OR = 0.927, 95% CI: 0.879–0.979) (Supplementary Table S6). The four methods of MR analysis were plotted for three statin use groups (Supplementary Figures S1-S3).

**TABLE 3:** Two sample MR analysis results of simvastatin use and pan-cancer *via* Fixed-effect inverse variance weighted (IVW_EF_) method.

Outcome	No. SNPs	β	SE_β_	OR (95% CI)	*p*
Bladder	39	0.000	0.004	1.000 (0.993-1.008)	1.000
Lung	39	−0.007	0.006	0.993 (0.983-1.004)	0.228
Bile duct	34	0.001	0.002	1.001 (0.997-1.005)	0.736
Liver cell	33	−0.001	0.001	0.999 (0.996-1.002)	0.371
Cervical	37	−0.004	0.005	0.996 (0.986-1.006)	0.419
Colorectal	39	0.008	0.008	1.008 (0.993-1.024)	0.309
Ovarian	39	0.003	0.007	1.003 (0.989-1.017)	0.686
Non-melanoma	39	−0.027	0.015	0.973 (0.945-1.002)	0.072
Melanoma	39	0.001	0.007	1.001 (0.988-1.014)	0.862
Prostate	39	0.539	0.233	1.714 (1.085-2.708)	**0.021**
Breast	39	0.163	0.293	1.176 (0.663-2.088)	0.579
Oesophagus	39	−0.004	0.003	0.996 (0.990-1.001)	0.132
Head and neck	39	−0.002	0.004	0.998 (0.991-1.005)	0.616

Abbreviation: MR: mendelian randomization; SNPs: single-nucleotide polymorphisms; SE: standard error; OR: odd ratio.

Bold values indicate statistical significance (*p*< 0.05).

**TABLE 4:** Two sample MR analysis results of rosuvastatin use and pan-cancer *via* Fixed-effect inverse variance weighted (IVW_EF_) method.

Outcome	No. SNPs	β	SE_β_	OR (95% CI)	*p*
Bladder	6	−0.011	0.047	0.989 (0.902-1.085)	0.814
Lung	6	−0.103	0.068	0.903 (0.790-1.031)	0.131
Bile duct	6	−0.053	0.025	0.948 (0.903-0.995)	**0.031**
Liver cell	6	−0.049	0.017	0.953 (0.921-0.985)	**0.005**
Cervical	6	−0.104	0.059	0.901 (0.803-1.011)	0.077
Colorectal	6	0.149	0.098	1.160 (0.958-1.405)	0.128
Ovarian	6	0.038	0.086	1.038 (0.877-1.229)	0.662
Non-melanoma	6	−0.084	0.186	0.920 (0.639-1.325)	0.653
Melanoma	6	0.009	0.080	1.009 (0.862-1.180)	0.913
Oesophagus	6	−0.002	0.036	0.998 (0.930-1.071)	0.958
Head and neck	6	−0.053	0.044	0.948 (0.870-1.033)	0.224

**Abbreviation**: MR: mendelian randomization; SNPs: single-nucleotide polymorphisms; SE: standard error; OR: odd ratio; CI: confidence interval.

Bold values indicate statistical significance (*p*< 0.05).

### Heterogeneity, pleiotropy, and sensitive analyses

To evaluate the heterogeneity of the results, Cochrane’s Q test was applied (Supplementary Tables S7-S9). There was substantial heterogeneity determined in lung cancer (*p* = 0.015), bile duct (*p* = 0.003), liver cell (*p* < 0.001), prostate (*p* < 0.001), and breast (*p* < 0.001) cancers of atorvastatin group *via* the IVW method. Additionally, significant heterogeneity results were also noticed in the bile duct, liver cell, prostate, and breast cancers (*p* < 0.001) of the simvastatin group and liver cell cancer (*p* = 0.013) in the rosuvastatin group. Therefore, the IVW_MRE_ method was alternatively performed to calculate the causal effect on these cancers (Table 5).

**TABLE 5:** Two sample MR analysis results of causality between three exposure groups and pan-cancer *via* multiplicative random effects IVW model method.

Category	No. SNPs	β	SE_β_	OR (95% CI)	*p*
**Atorvastatin**					
Lung cancer	22	−0.012	0.018	0.988 (0.954-1.023)	0.501
Bile duct cancer	21	−0.009	0.007	0.991 (0.977-1.005)	0.213
Liver cell cancer	21	−0.007	0.007	0.993 (0.979-1.006)	0.274
Prostate cancer	22	0.878	0.995	2.405 (0.342-16.902)	0.378
Breast cancer	21	0.372	1.173	1.451 (0.146-14.461)	0.751
**Simvastatin**					
Bile duct cancer	34	0.001	0.003	1.001 (0.994-1.007)	0.817
Liver cell cancer	33	−0.001	0.003	0.999 (0.993-1.004)	0.619
Non-melanoma	39	−0.027	0.024	0.973 (0.929-1.019)	0.250
Prostate cancer	39	0.539	0.566	1.714 (0.565-5.202)	0.341
Breast cancer	39	0.163	0.552	1.176 (0.399-3.468)	0.768
**Rosuvastatin**					
Liver cell cancer	6	−0.049	0.029	0.953 (0.899-1.009)	0.097

**Abbreviation**: MR: mendelian randomization; SNPs: single-nucleotide polymorphisms; SE: standard error; OR: odd ratio; CI: confidence interval.

The significant causal effects disappeared among atorvastatin use and liver cell cancer (OR = 0.993, 95% CI: 0.979–1.006, *p* = 0.274), simvastatin use and prostate cancer (OR = 1.714, 95% CI: 0.565–5.202, *p* = 0.341), rosuvastatin use and liver cell cancer (OR = 0.953, 95% CI: 0.899–1.009, *p* = 0.097), respectively.

Moreover, there was no horizontal pleiotropy detected in the three exposure groups (*p* > 0.05), which indicated that the results were not influenced by the potential confounding pathways, and the results were rational and robust (Supplementary Tables S7-S9). The leave-one-out analysis was conducted for each cancer outcome to assess the stability of results in the atorvastatin (Supplementary Figure S1), simvastatin group (Supplementary Figure S2), and rosuvastatin group (Supplementary Figure S3). Similarly, the results in the Leave-one-out analysis were consistent with that in the primary IVW analysis. Furthermore, the funnel plot was made to present the distribution balance of single SNP effects in three groups (Supplementary Figures S1-S3). The original data of the tests were included in Supplementary Tables S10-S15.

## Discussion

To the best of our knowledge, this is the only one of few studies on evaluating the genetic association between stain use and pan cancer risk. Detailly, with three frequently used statins and thirteen cancer types involved, this two-sample MR analysis did not show remarkable causality between statin uses and pan-cancer prevention. For the exposure to atorvastatin use, statistically significant relationships were only demonstrated between atorvastatin and colorectal cancer risk (OR = 1.041, by IVW_FE_ method, OR = 1.086, by weighted median; OR = 1.101, by weighted mode, respectively). Our results were consistent with one recent prospective study explored by Zhang et al. (Zhang et al., 2022). Within 100,300 women and 47,991 men in the Nurses’ Health Study and Health Professionals Follow-Up Study, they demonstrated that there was no significant beneficial effect of statins in decreasing colorectal cancer risk but a higher risk of developing colon cancer was observed in long-term statin use (>15 years, hazard ratio (HR) = 1.85, 95% CI: 1.31–2.61). One recent study with a new target trial design (refers to a more rational analysis method in evaluating the case-control study) was conducted by Dickerman et al. (Dickerman et al., 2020). With linked electronic health records of 752 469 UK adults, the results did not find any significant association between long-term statins use (>5 years) and colon cancer risk (HR = 0.90, 95% 0.71–1.12). Interestingly, studies evaluating the role of stain use on colon cancer survival were consistent (Voorneveld et al., 2017; Yokomichi et al., 2017; Pourlotfi et al., 2022). Notably, as reported by Voorneveld et al. (Voorneveld et al., 2017), statin use after diagnosis was significantly associated with reduced risk of overall and cancer-specific mortality (relative risk (RR) = 0.67, 95% CI: 0.51–0.87, RR = 0.66, 95% CI: 0.49–0.89, respectively). Their tissue microarray results revealed that the protective role of colon cancer survival was remarkably associated with intact BMP signaling pathways, regardless of the K-RAS mutation status. The disparity role of statin uses in colon cancer development and survival indicated the complex interaction of statins and colon cancer was presented. Thus, the deepening of exploring the underlying biological mechanisms is warranted in future works.

Additionally, our study observed a slightly protective effect of rosuvastatin use on bile duct cancer (OR = 0.948, by IVW_FE_ method) prevention. Reviewing recent literature, the conclusions for bile duct cancer prevention with statin use were in conflict. Liu et al. (Liu et al., 2019) reported that statin use was associated with a nearly 12% lower risk of bile duct cancer (OR = 0.88, 95% CI: 0.79–0.98), compared with non-use of statins. Meanwhile, the latest meta-analysis based on eight observational studies also supported this correlation (Cheung et al., 2023). Conversely, from the same European population, the team of Tran et al. (Tran et al., 2020) yielded the benefits role of statin use in liver cancer prevention (OR = 0.48, 95% CI: 0.24–0.94) but not in bile duct cancer (HR = 1.09, 95% CI: 0.45–2.64). In Tran’s work, during the univariate analysis, statin use was associated with an increased risk of developing liver cell cancer (HR = 1.30, 95% CI: 0.80–2.10). However, after adjusting for numerous confounders, the hazard ratio became significant (HR = 0.48, 95% CI: 0.24–0.94). It indicates that the confounders played an important role during the analysis. For this reason, the ignored but pivotal confounders, which were not adjusted during analysis, could hide the real correlations between the statin use and cancer prevention. Besides, findings based on the Asian population, statins use might decrease the liver cell cancer risk by 59% in patients with chronic liver diseases and by 22% in patients without chronic liver diseases (Lai et al., 2013). Although the supplementary MR analyses in our study revealed the statistically significant causality between statin use and liver cell cancer, the protective efforts of stains were limited. And the significance disappeared after changing the analysis method to IVW_MRE_ owing to the heterogeneity test results. Hereby, our study did not find remarkably decreased liver cell cancer risk in the statin-use population at a genetic level. Future genetic analysis with more comprehensive and latest GWAS datasets could get more robust evidence on this topic.

Regarding skin cancer, especially in terms of non-melanoma cancer, no significant causality was determined during the MR analysis, regardless of the subtype of statins or MR methods. Similar to our findings, one earlier meta-analysis with robust evidence from 29 studies also supported our negative results (Li et al., 2014). By contrast, with a detailed sub-analysis, Arnspang et al. demonstrated there was a positive correlation between simvastatin (OR = 1.10, 95% CI:1.01–1.19) and fluvastatin (OR = 1.59, 95% CI: 1.17–2.16) and basal cell skin cancer (Arnspang et al., 2015). Based on our results and previous studies, limited evidence could support stain use for general skin cancer prevention.

Moreover, there was no causality was detected in statins use and female cancers in our results and we partially validated the results from one recent meta-analysis conducted by Wang et al. (Wang et al., 2019). In their study, statin use and the duration had no impact on the risk of ovarian cancer or endometrial cancer. Studies evaluating the association between statin use and cervical cancer risk were scarce. Nevertheless, one study preliminary evaluated the beneficial role of stain use in the prognosis of cervical cancer (Song et al., 2017). While the results were encouraging that the stains use could remarkably prolong the survival of stages IB to IV patients, the evidence was limited owing to the relatively small sample size and short follow-up time). For these reasons, future well-designed studies with large-scale patients might help confirm our findings on cervical cancer.

During the past few years, the role of statin uses in prostate cancer raised wide concerns (Tan et al., 2011; Alfaqih et al., 2017; Longo et al., 2022). Notably, prostate cancer patients on statins were noticed to have a lower rate of high grade, lower prostate volume, and lower prostate-specific antigen, compared with the non-statins population (Tan et al., 2011). In recent comprehensive review described, the possible mechanisms by which statins could induce the anticancer effect in multiple ways including but not limited to cholesterol-mediated pathways, and apoptosis in prostate cancer cells (Alfaqih et al., 2017; Longo et al., 2022). In contrast, our results showed there was a positive causality between simvastatin use and prostate cancer risk before changing the MR methods (OR = 1.714, 95% CI: 1.085–2.708). By using the IVW_MRE_ method, this association did not exist. Thus, the MR analysis could not support the beneficial hypothesis of statin use on prostate cancer.

On the other hand, there was a trend to be significant in atorvastatin use and head and neck cancer risk (*p* = 0.052). And evidence from different ethnicity-based studies also demonstrated the inverse correlation between statins and head and neck cancer risk (Kao et al., 2019; Getz et al., 2021). Interestingly, evidence from the large-scale SEER-Medicare linked datasets highlighted the protective role of statin use in the survival outcome of head and neck cancer patients (Gupta et al., 2019). The optimal findings among statin use and the prolonged survival probabilities in cancer survivors might be due to the unique effects of statin on promoting the apoptosis of cancer cells and inhibiting the cell proliferation as well as the invasion in cancer related metabolism (Wang et al., 2000; Wong et al., 2002; Chan et al., 2003; Nübel et al., 2004; Kidera et al., 2010). Furthermore, recent clinical and preclinical studies also demonstrated the statin could be the radio-sensitizers in cancer therapy (Wei et al., 2022). Thus, these findings help to reveal the potential mechanisms of the beneficial role of statin use in cancer therapy (Duarte et al., 2021).

As for other frequent solid tumors, like breast cancer, lung cancer, bladder cancer, as well as oesophageal cancer, our MR analysis results confirmed the results in previous studies that there was no association between statin use and the risk of these mentioned cancers (Setoguchi et al., 2007; Kuo et al., 2012; Undela et al., 2012; Chan et al., 2013; Chan et al., 2014). Interestingly, however, recent works suggested that statins might improve the survival probabilities in these cancer types like the results observed in liver cell cancer (Alexandre et al., 2016; Manthravadi et al., 2016; Richard et al., 2017; Borgquist et al., 2019; Chen et al., 2019). Emerging evidence even revealed that statins users appeared to have substantially better survival outcomes than nonusers in different observational studies. However, some scholars speculated that the inverse association between statin use and cancer mortality might be owing to data collection and selection bias, and immortal-time bias (Emilsson et al., 2018). Thereby, the confirmatory works done by Emilsson et al. (Emilsson et al., 2018) demonstrated that the bias existing in previous observational studies could be reduced by using the inverse-probability weighting method in data censoring. Compared with the positive findings in previous observational studies, neither 3-year cancer-specific survival nor overall survival was improved in patients who started the statin therapy within 6 months during postdiagnosis (Emilsson et al., 2018). Nevertheless, although this emulated trial yielded the negative influence of selection bias on evaluating the statin use and cancer survival, it should not discourage the enthusiasm for conducting trials of statins in cancer prevention and survival since statin repurposing was expected to be a more cost-effective way compared to some other newly modalities of cancer treatment (Ahern et al., 2018; Chan et al., 2018).

There are some strengths of our study that need to be mentioned. First, to our knowledge, this is the first comprehensive analysis in evaluating the three different statins use and pan-cancer risk *via* the two-sample MR analysis, which could provide more insightful evidence for future investigation. Second, compared with previous clinical observational or case-control studies, the GWAS datasets maintain stability in the same ancestry population. The data stability could help to reduce the bias during the outcome comparisons. Third, there is no horizontal pleiotropy observed during the MR analysis and the leave-one-out analysis proved the stability of the MR analysis. While there are some heterogeneities existed in several cancers, the changed MR method could help to reduce this bias.

Nonetheless, several limitations are existed in this study, which should be noticed in the interpretation and generalization of our findings. First, although the study population in this work eliminates the race discrepancy, it is unknown whether the findings could be generalized to other different race and regions. Thus, GWAS studies from different regions could provide more robust evidence on statin use and cancer risk. Moreover, the GWAS data on statins and cancer are updated periodically, and future datasets with comprehensive SNPs results could provide more significant results. While our results only determine a slightly beneficial role of statin use in some particular cancers, future well-designed double-blind randomized controlled trials with matched participants enrollment, the same dosage of statin use, the same duration of medication of statin therapy, and long-term follow-up information, are warranted to validate these findings and provide more robust evidence in evaluating the feasibility of lipid-lowering therapy in the anti-cancer process. Regarding the nonrandomized trials, subgroup analyses can be considered in specific populations with a high risk of cancer occurrence, which could assist clinicians to find the most suitable population to receive the statin therapy for cancer-related chemoprevention.

## Conclusion

Generally, results from this large-scale pan-cancer MR analysis, the statistical significance causalities are only observed in atorvastatin use and colorectal cancer and rosuvastatin use and bile duct cancer in European ancestry population. For this reason, we could not provide sufficient evidence to support that statin use could remarkably reduce the risk of different cancers. The role of statin use in cancer prevention remains debatable and there is still a long way to go before considering devoting the stains as chemo-preventive drugs into clinical practice. On the one hand, there are still existing disparities between remarkably anticarcinogenic effects in preclinical models and negative results in real-world observational studies. On the other hand, the potential confounding factors that existed in observational studies could also overlap the real influence of statin use in cancers. Therefore, future well-designed double-blind randomized controlled trials are warranted to validate the existing correlations we determined.

## Data Availability

The original contributions presented in the study are included in the article/Supplementary Material, further inquiries can be directed to the corresponding authors.
