# Supplementary material for: Assessing the role of lipid-lowering therapy on multi-cancer prevention: A mendelian randomization study
Source: Front Pharmacol. 2023 Apr 19;14:1109580. doi: 10.3389/fphar.2023.1109580 (PMC10154601; doi:10.3389/fphar.2023.1109580)

Supporting information

Assessing the Role of Lipid-lowering Therapy on Multi-Cancer Prevention: A Mendelian Randomization Study

Running title: Statins use and pan-cancer prevention

Yu Min [a#], Xiaoyuan Wei [b#], Zheran Liu [a#], Zhigong Wei [a], Yiyan Pei [a], Ruidan Li[a], Jing Jin[a], Yongllin Su [d], Xiaolin Hu [e*], Xingchen Peng [a*]

a. Department of Biotherapy and National Clinical Research Center for Geriatrics, Cancer Center, West China Hospital, Sichuan University, Sichuan, China.
b. Department of Head and Neck Oncology, Department of Radiation Oncology, Cancer Center, and State Key Laboratory of Biotherapy, West China Hospital, Sichuan University, Sichuan, China.
c. Department of Rehabilitation, Cancer Center, West China Hospital, Sichuan University, Sichuan, China
d. West China School of Nursing, West China Hospital, Sichuan University, Sichuan, China.

#Yu Min, Xiaoyuan Wei, and Zheran liu contributed equally to this work.

*Corresponding Authors:

Prof. Xingchen Peng is to be contacted at the Department of Biotherapy, Cancer Center, West China Hospital, Sichuan University, Chengdu 610041, Sichuan, China. E-mail address: pxx2014@163.com.

Prof. Xiaolin Hu is to be contacted at West China School of Nursing, West China Hospital, Sichuan University, Chengdu 610041, Sichuan, China. E-mail address: huxiaolin@wchscu.cn.

Figure S1. (A) The singly SNP estimated the causal effect of atorvastatin use on pan-cancer risks; (B) the Leave-one-out analysis in estimating the causal effect of atorvastatin use on pan-cancer risks; (C) The four methods of MR test; (D) The scatter diagram for showing the tendency of dispersion in each cancer.

Bladder cancer

A

B

C

D

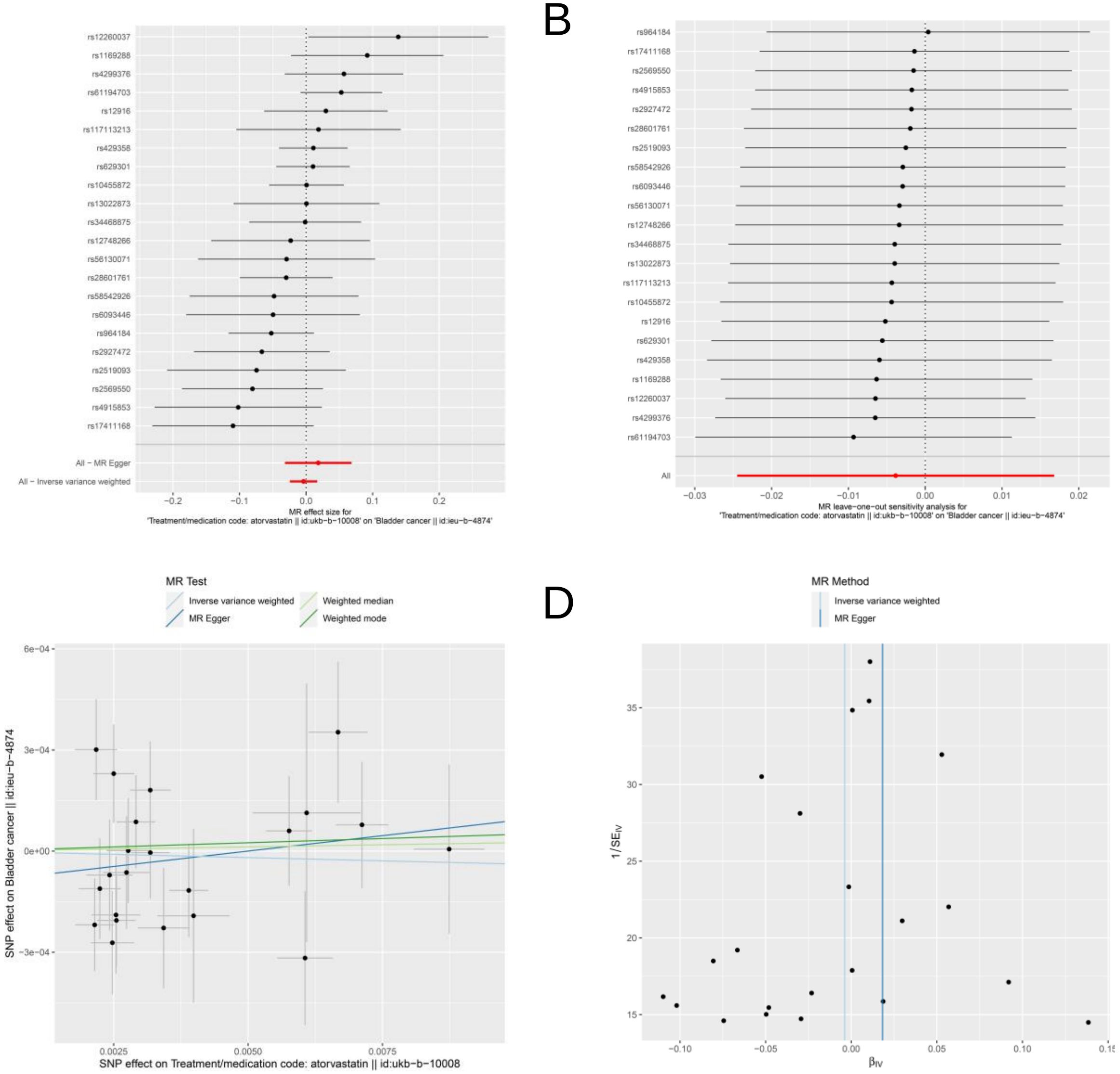

Lung cancer

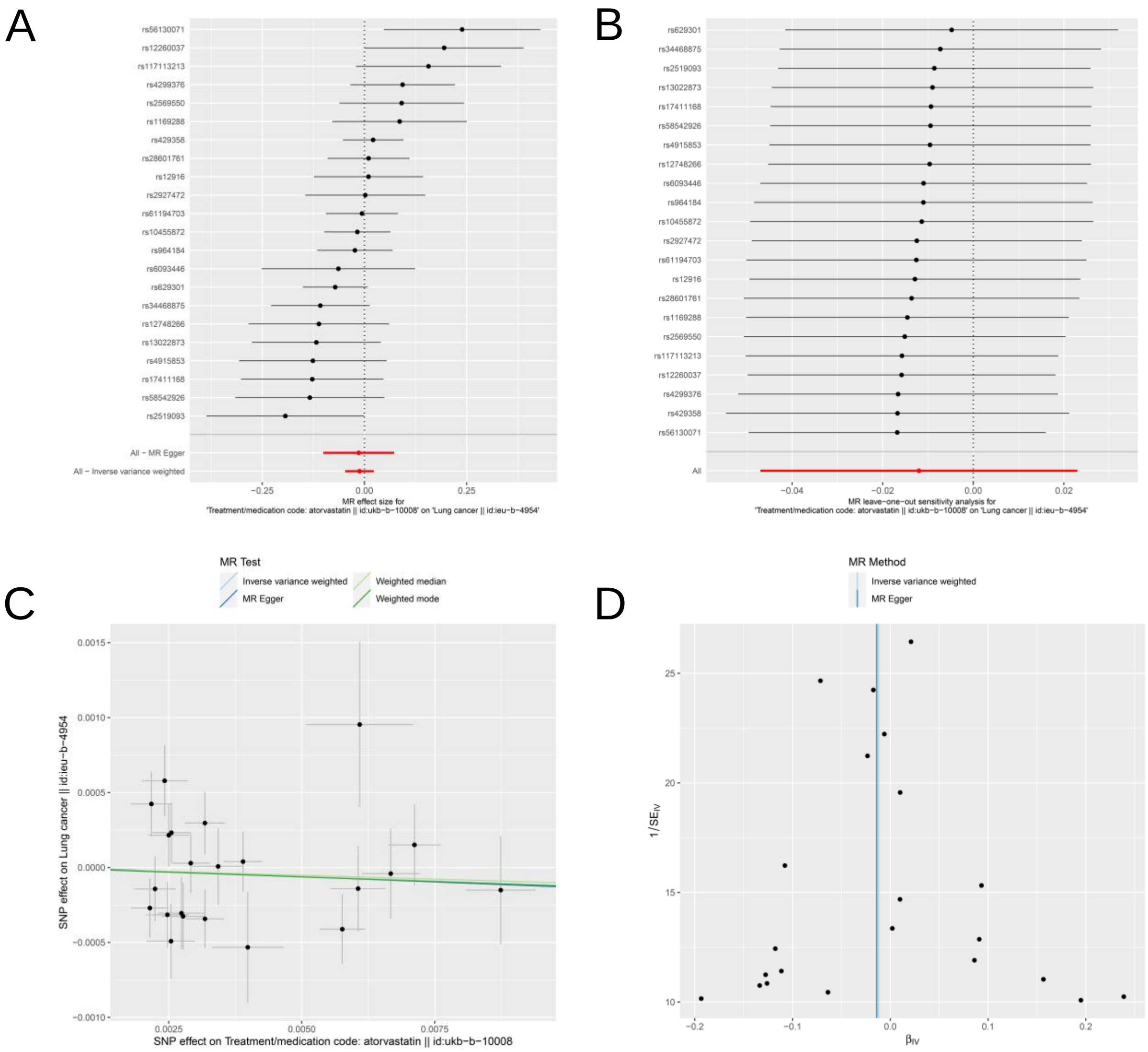

A
B
C
D
MR effect size for
'Treatment/medication code: atorvastatin || id:ukb-b-10008' on 'Lung cancer || id:ieu-b-4954'
All – MR Egger
All – Inverse variance weighted
MR leave-one-out sensitivity analysis for
'Treatment/medication code: atorvastatin || id:ukb-b-10008' on 'Lung cancer || id:ieu-b-4954'
All
MR Test
Inverse variance weighted
MR Egger
Weighted median
Weighted mode
SNP effect on Treatment/medication code: atorvastatin || id:ukb-b-10008
SNP effect on Lung cancer || id:ieu-b-4954
MR Method
Inverse variance weighted
MR Egger

Bile ductal cancer

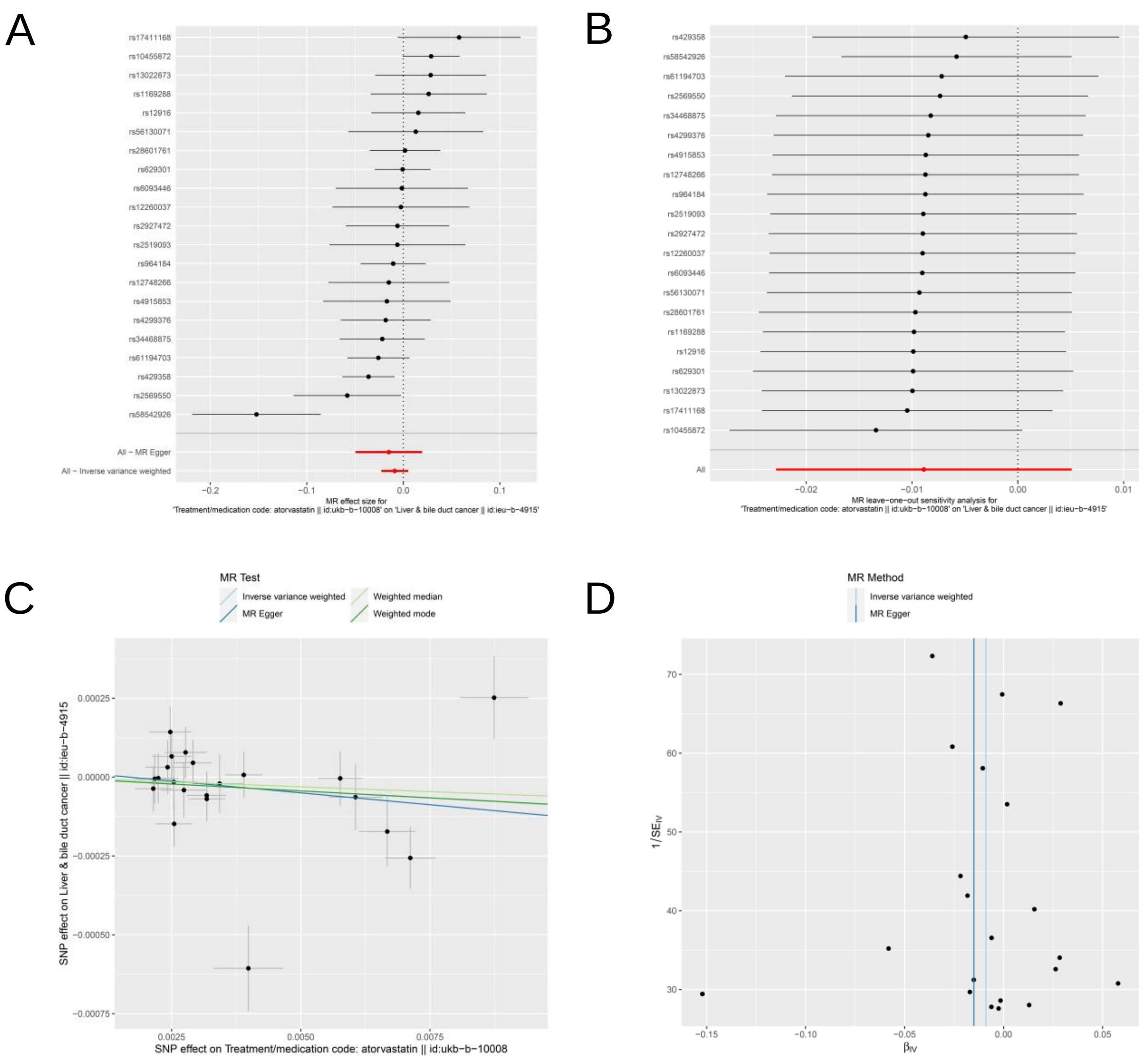

Liver cancer

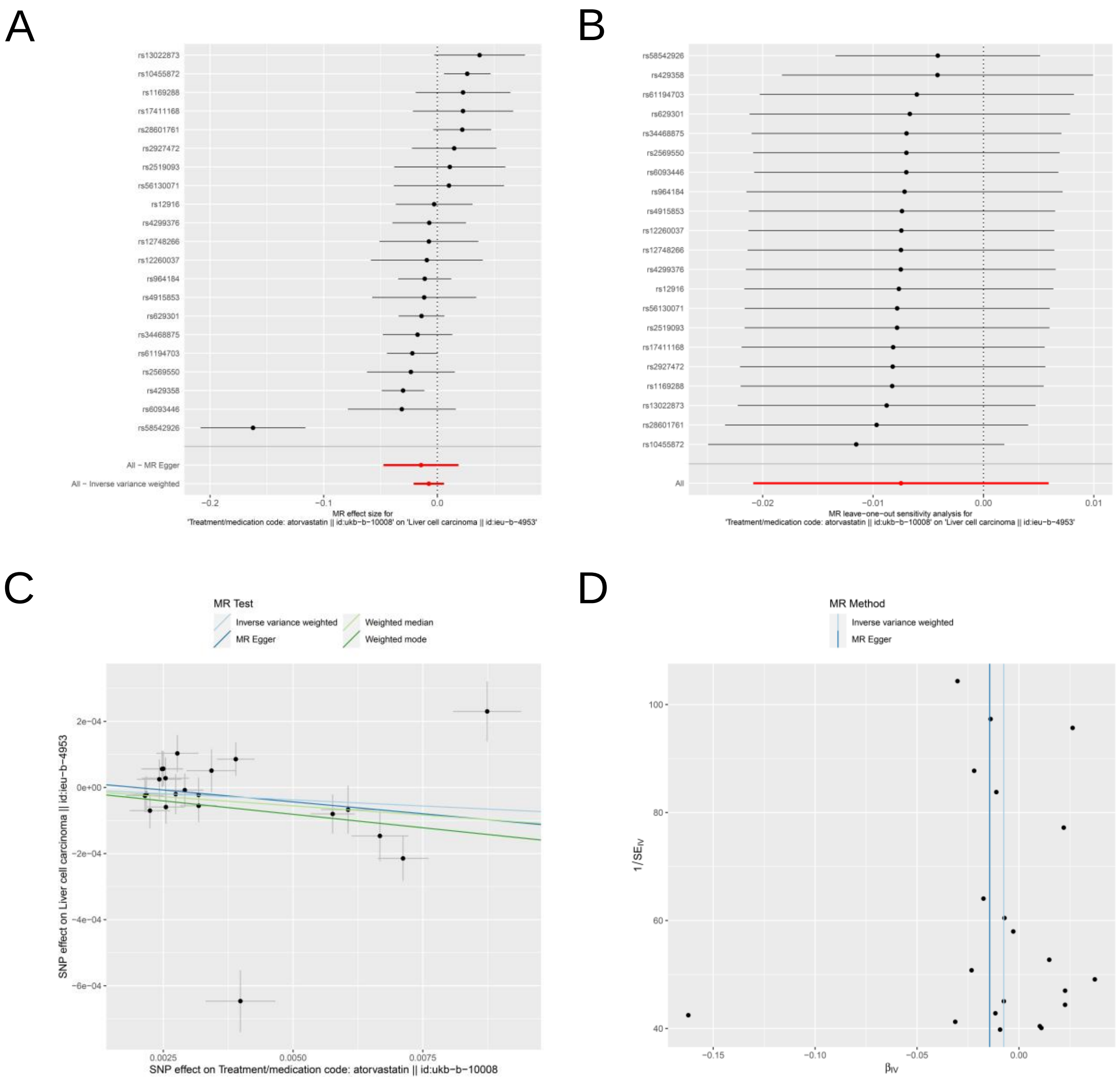

Cervical cancer

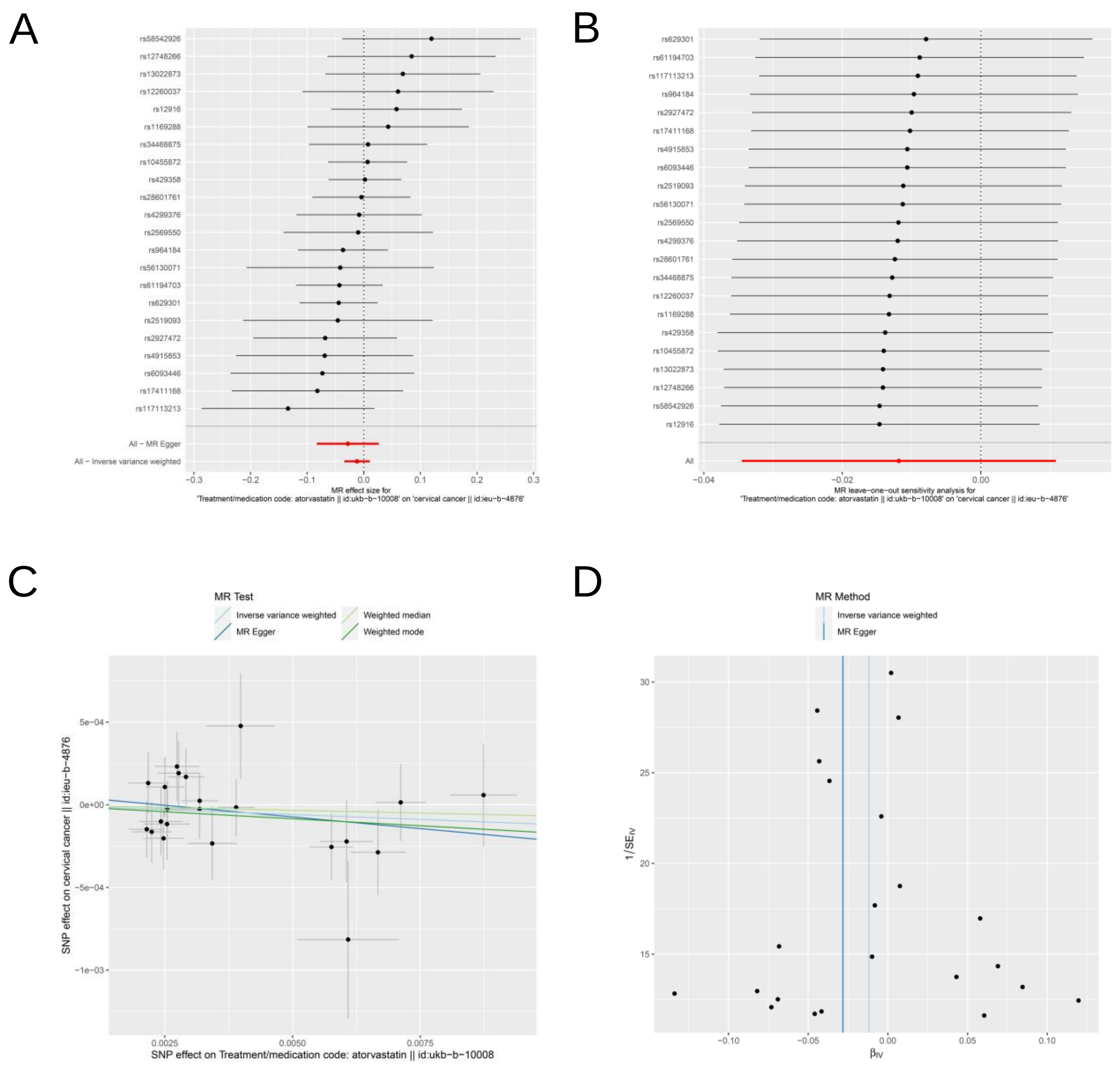

Colorectal cancer

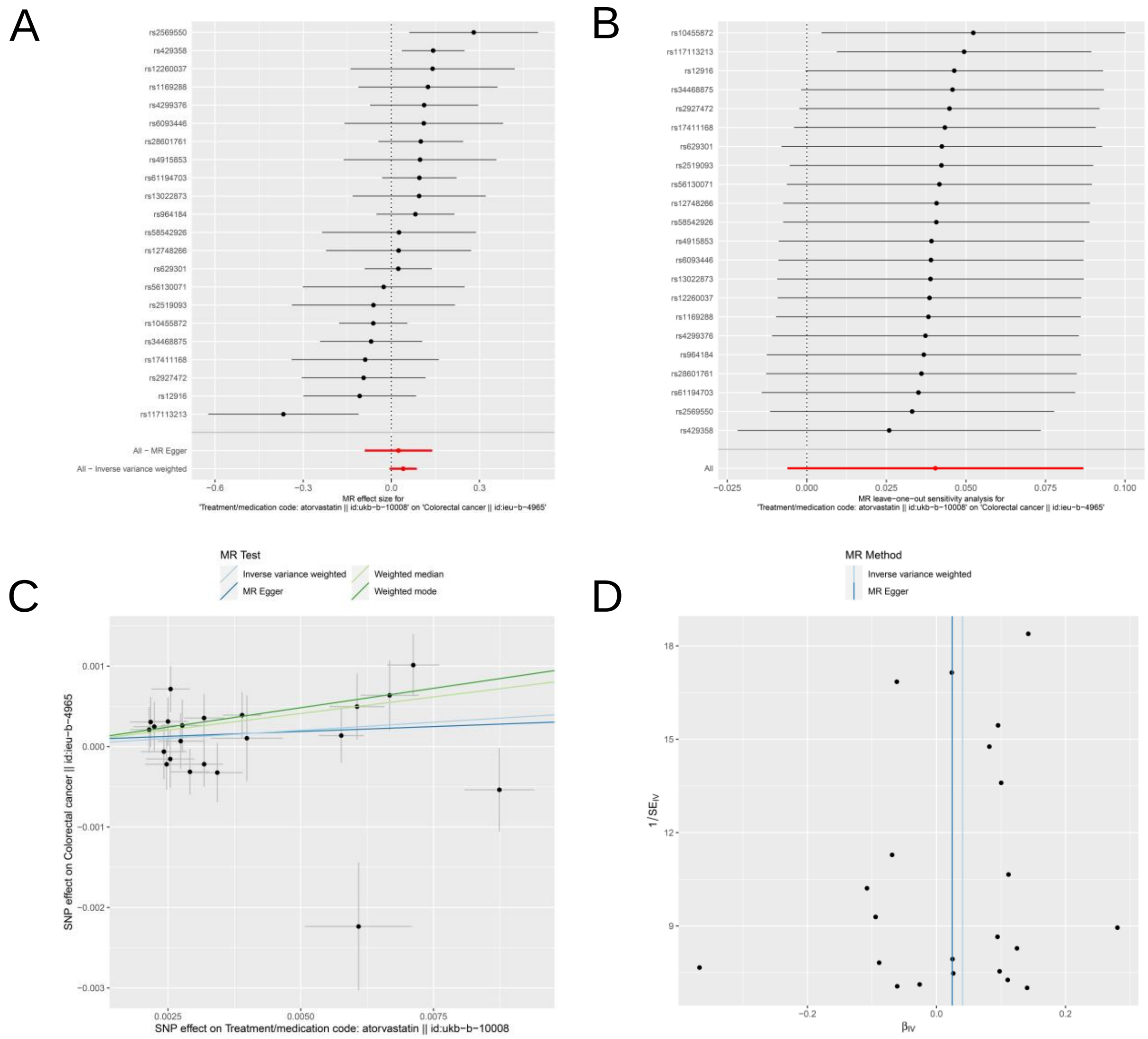

Ovarian cancer

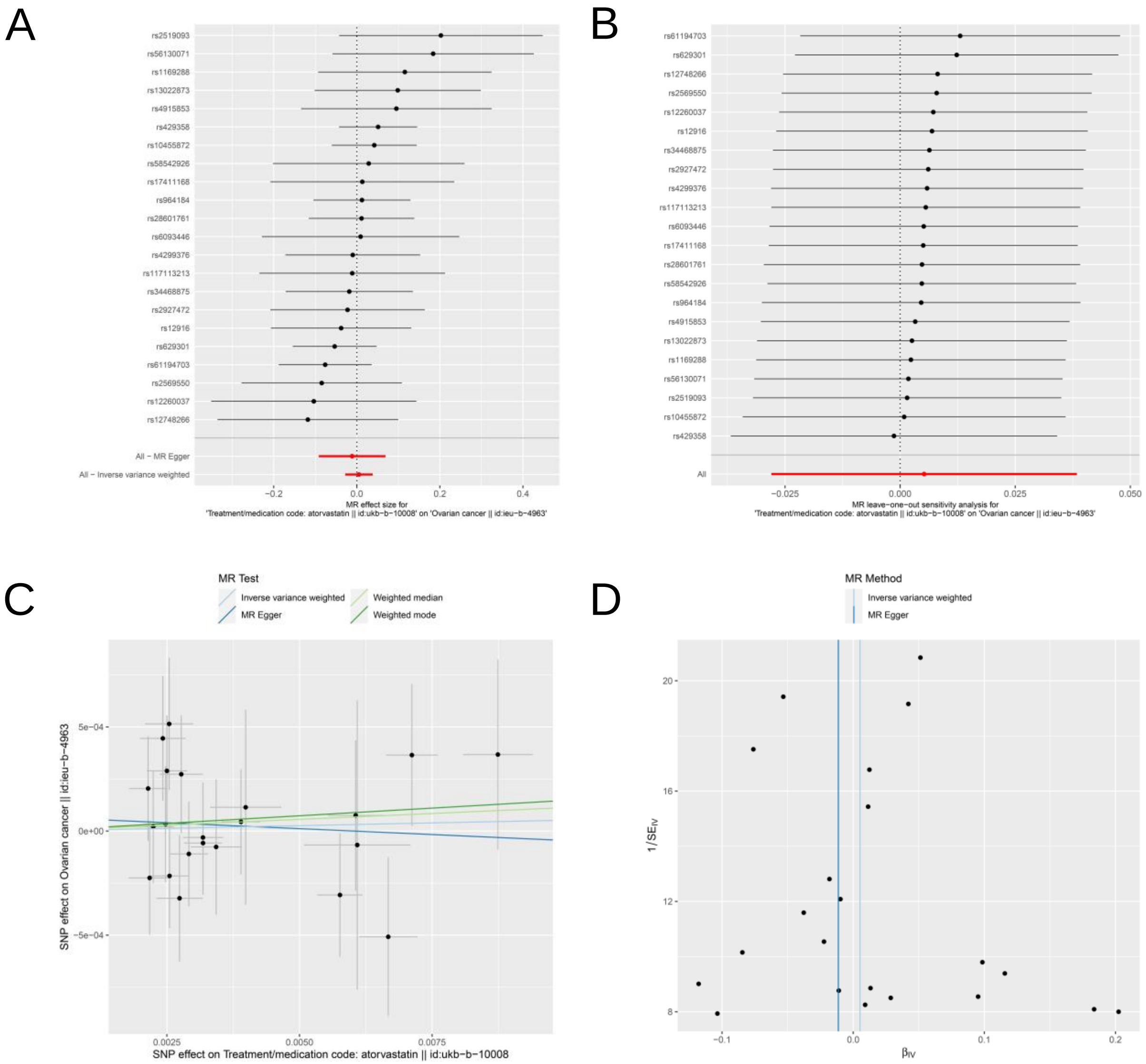

Non-melanoma

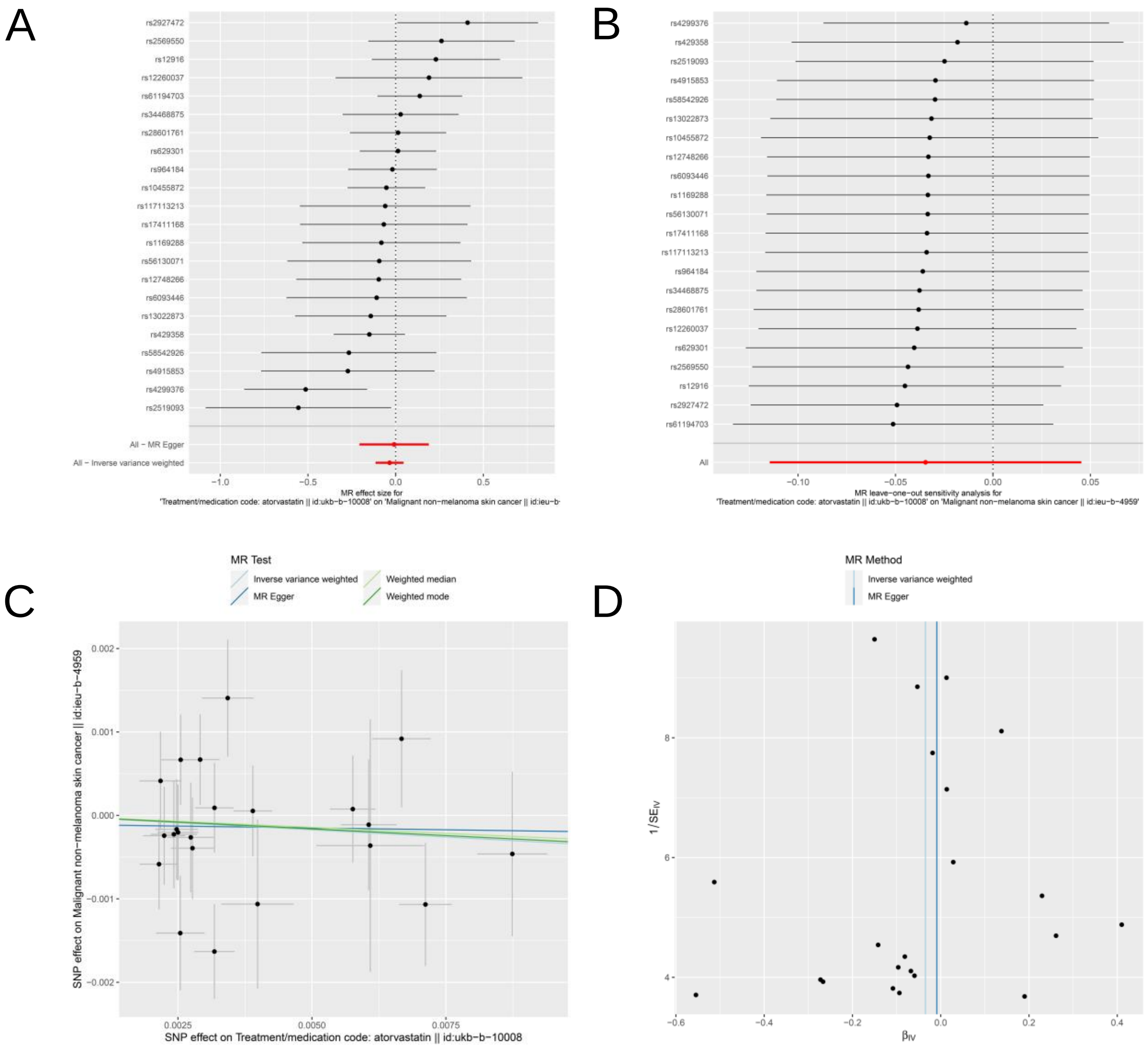

Melanoma

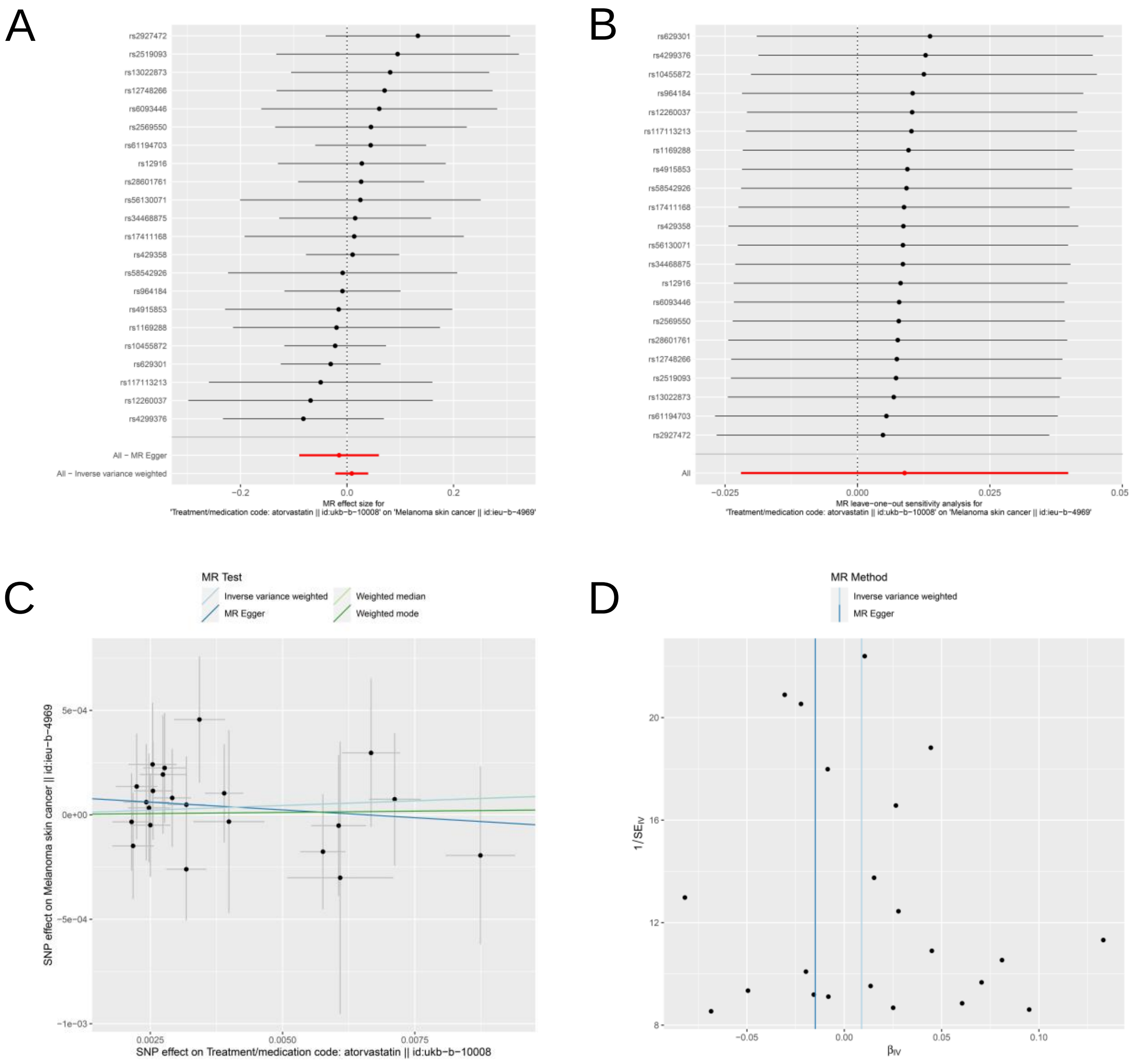
A
B
C
D
All − MR Egger
All − Inverse variance weighted
MR effect size for
'Treatment/medication code: atorvastatin || id:ukb−b−10008' on 'Melanoma skin cancer || id:ieu−b−4969'
All
MR leave−one−out sensitivity analysis for
'Treatment/medication code: atorvastatin || id:ukb−b−10008' on 'Melanoma skin cancer || id:ieu−b−4969'
MR Test
Inverse variance weighted
MR Egger
Weighted median
Weighted mode
SNP effect on Melanoma skin cancer || id:ieu−b−4969
SNP effect on Treatment/medication code: atorvastatin || id:ukb−b−10008
MR Method
Inverse variance weighted
MR Egger

Prostate cancer

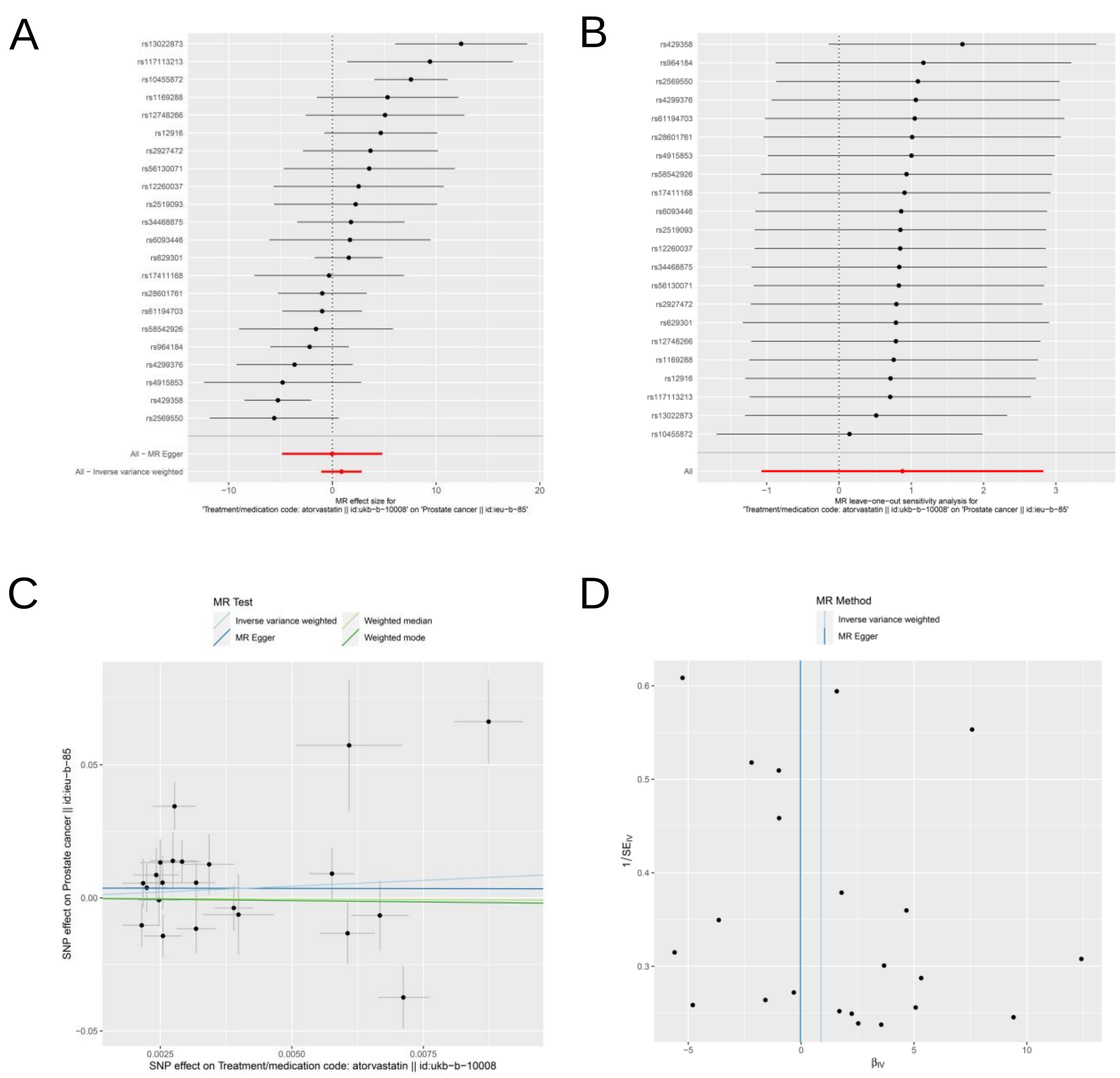

Breast cancer

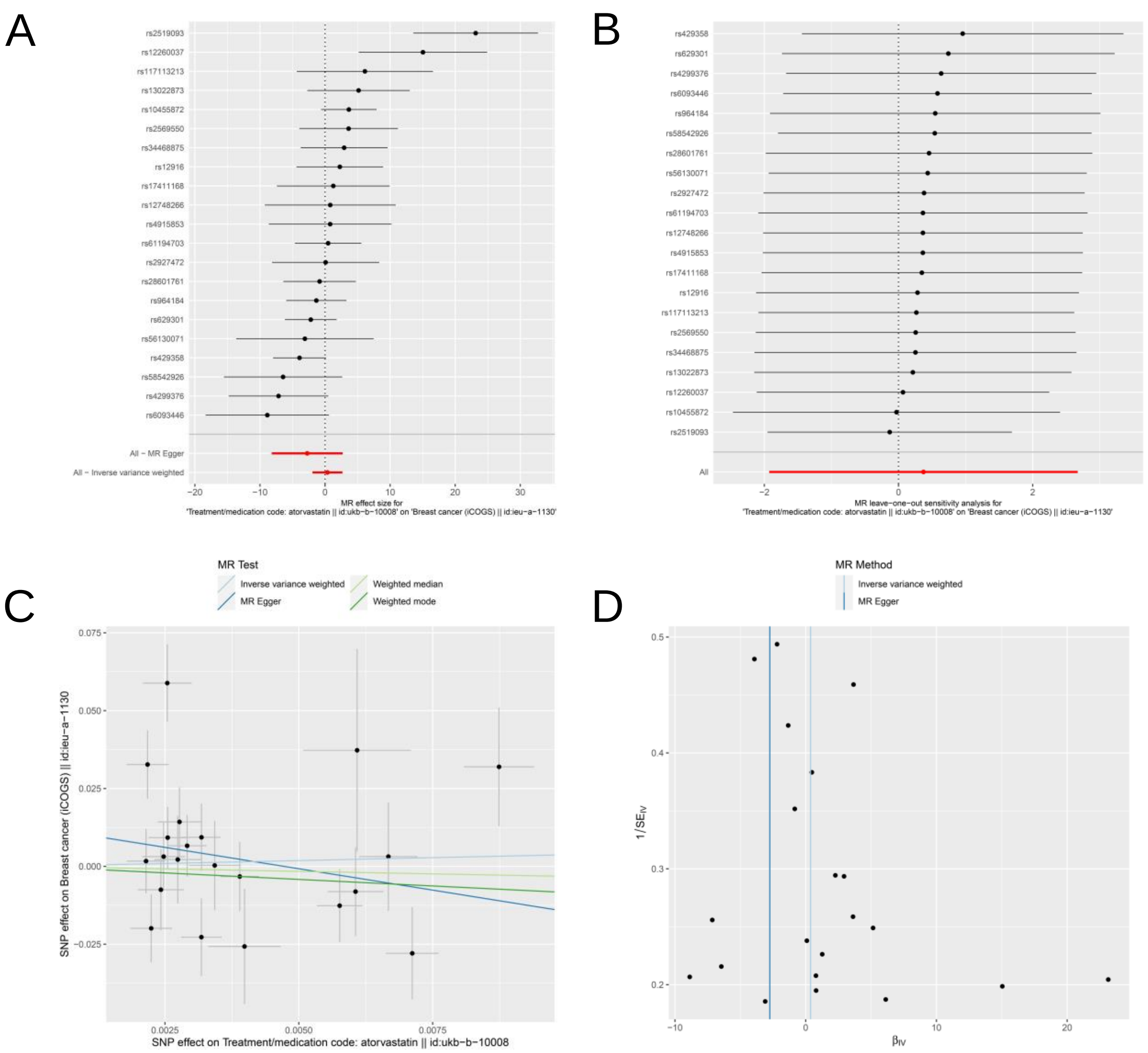

esophageal cancer

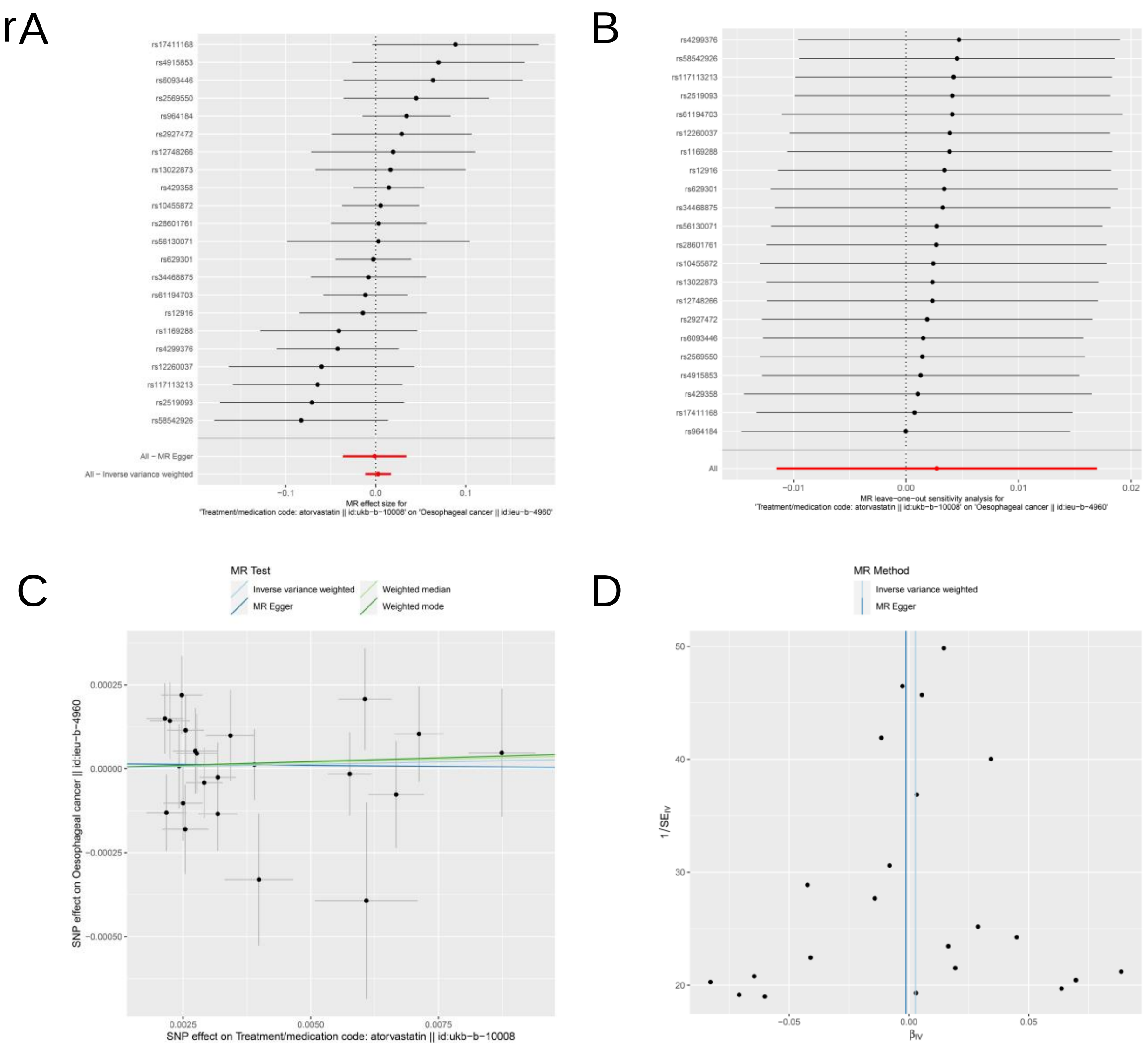

Head and neck Cancer

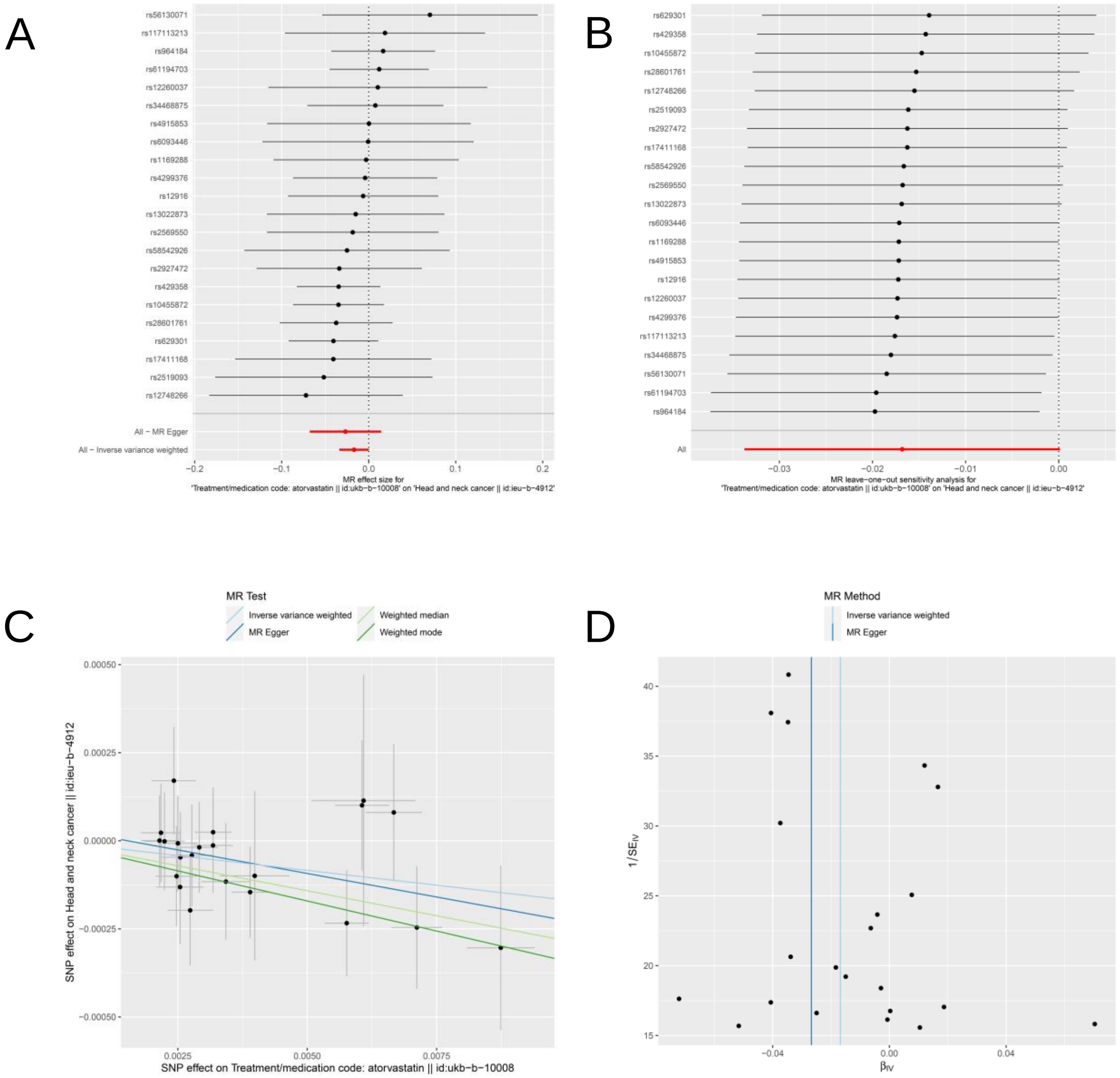

Figure S2. (A) The singly SNP estimated the causal effect of simvastatin use on pan-cancer risks; (B) the Leave-one-out analysis in estimating the causal effect of atorvastatin use on pan-cancer risks; (C) The four methods of MR test; (D) The scatter diagram for showing the tendency of dispersion in each cancer.

Bladder cancer

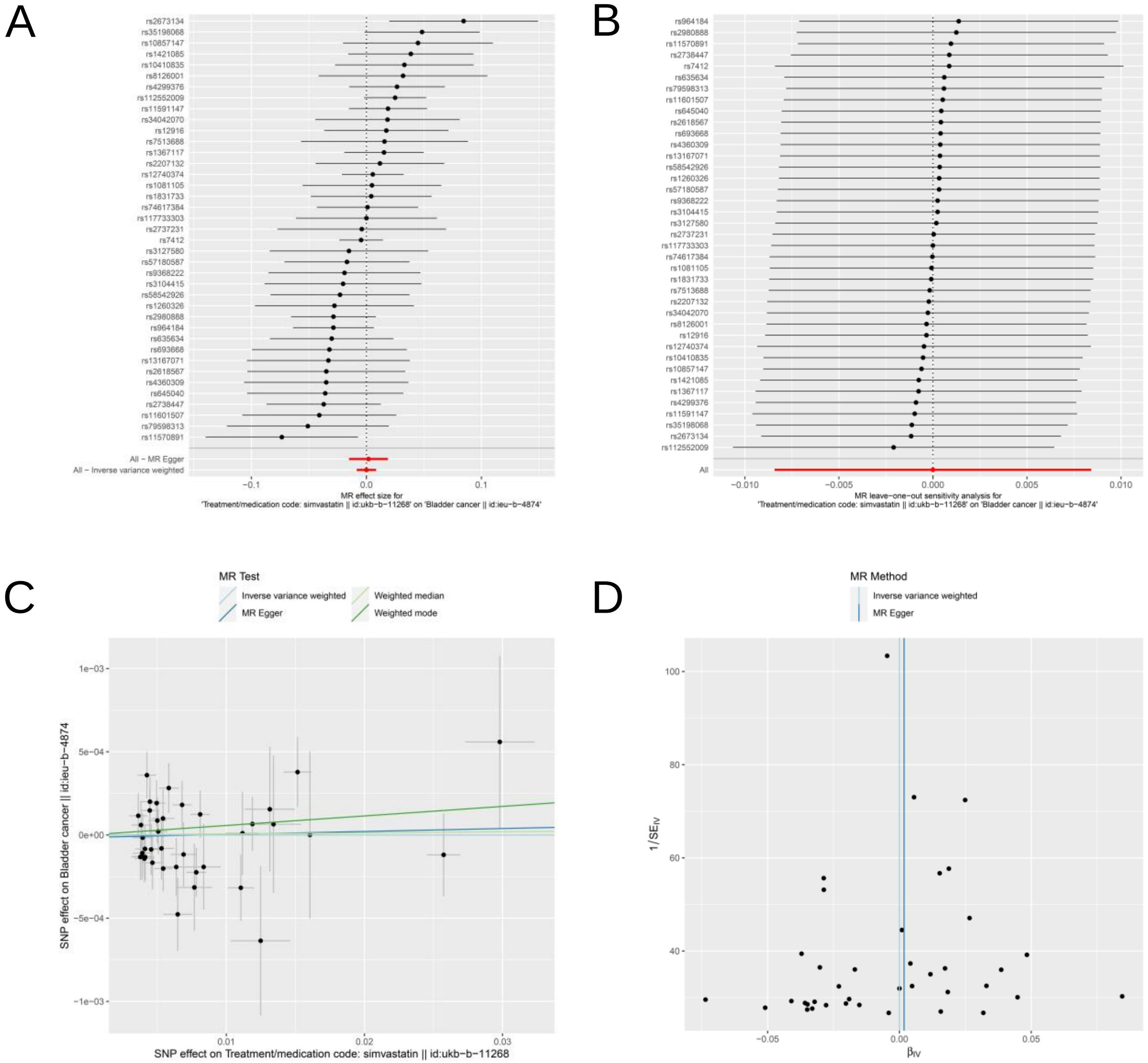

Lung cancer

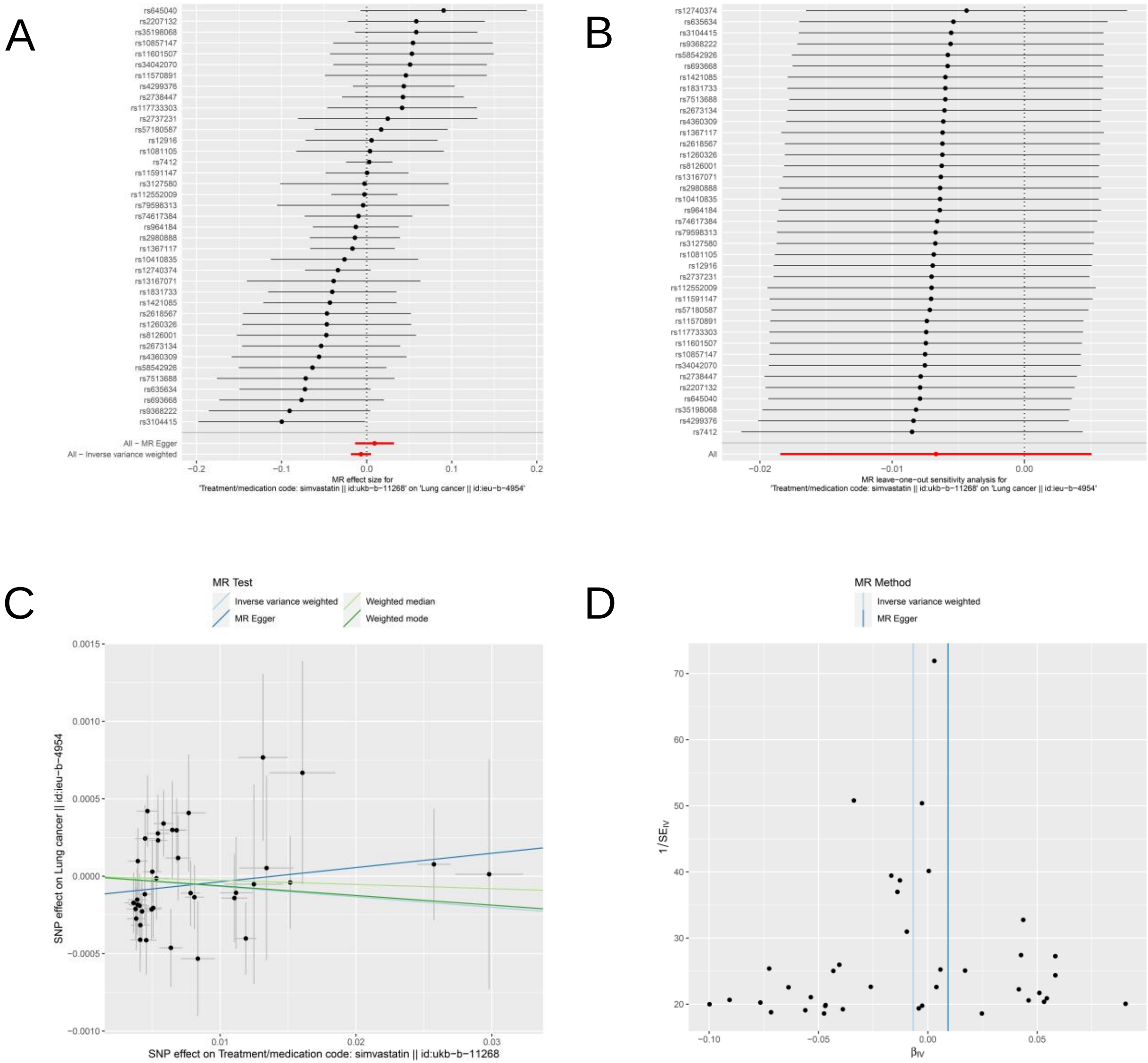
A
B
C
D
All – MR Egger
All – Inverse variance weighted
MR effect size for
'Treatment/medication code: simvastatin || id:ukb-b-11268' on 'Lung cancer || id:ieu-b-4954'
All
MR leave-one-out sensitivity analysis for
'Treatment/medication code: simvastatin || id:ukb-b-11268' on 'Lung cancer || id:ieu-b-4954'
MR Test
Inverse variance weighted
MR Egger
Weighted median
Weighted mode
SNP effect on Lung cancer || id:ieu-b-4954
SNP effect on Treatment/medication code: simvastatin || id:ukb-b-11268
MR Method
Inverse variance weighted
MR Egger
1/SE_IV
β_IV

Bile ductal cancer

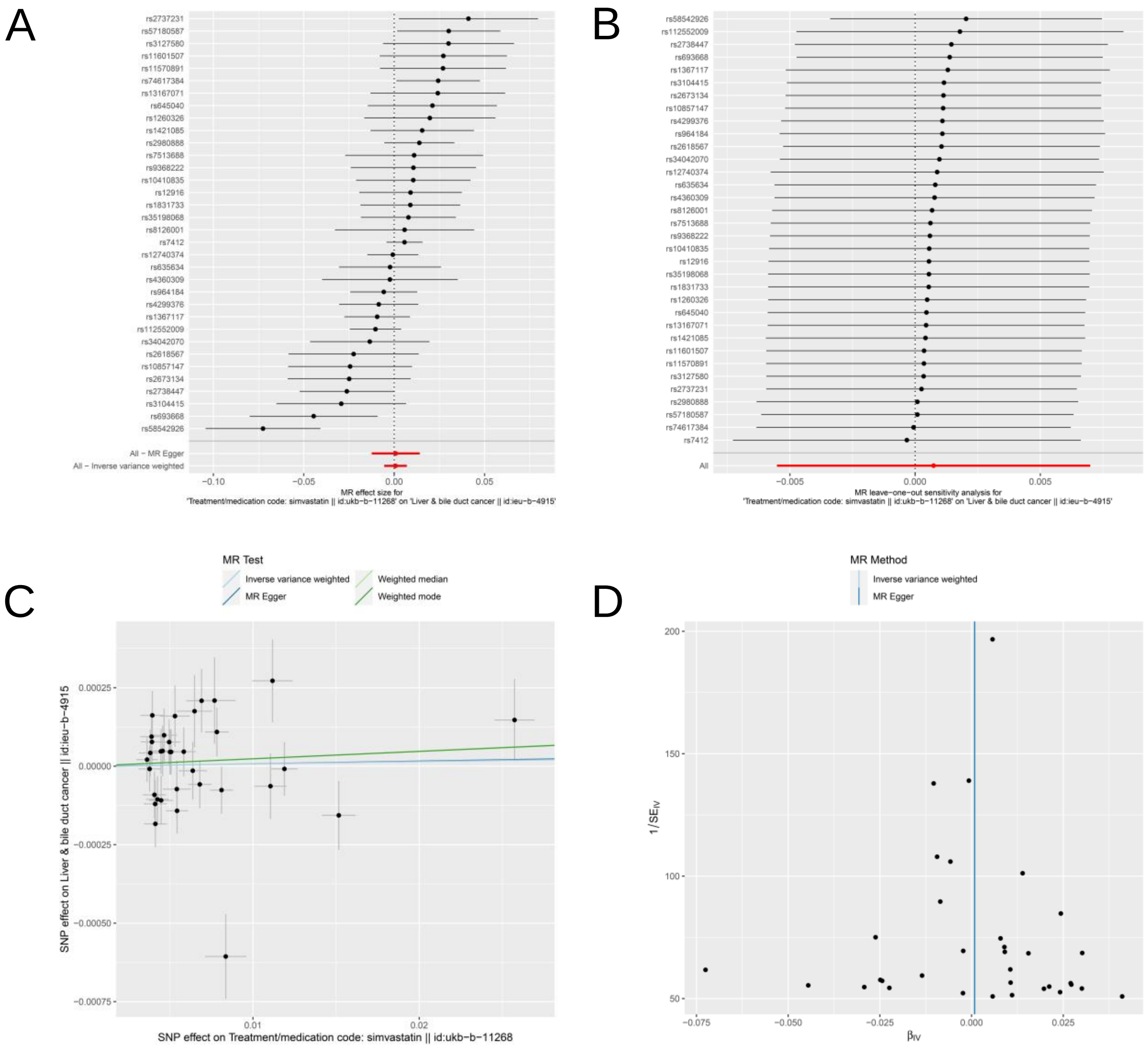

Liver cancer

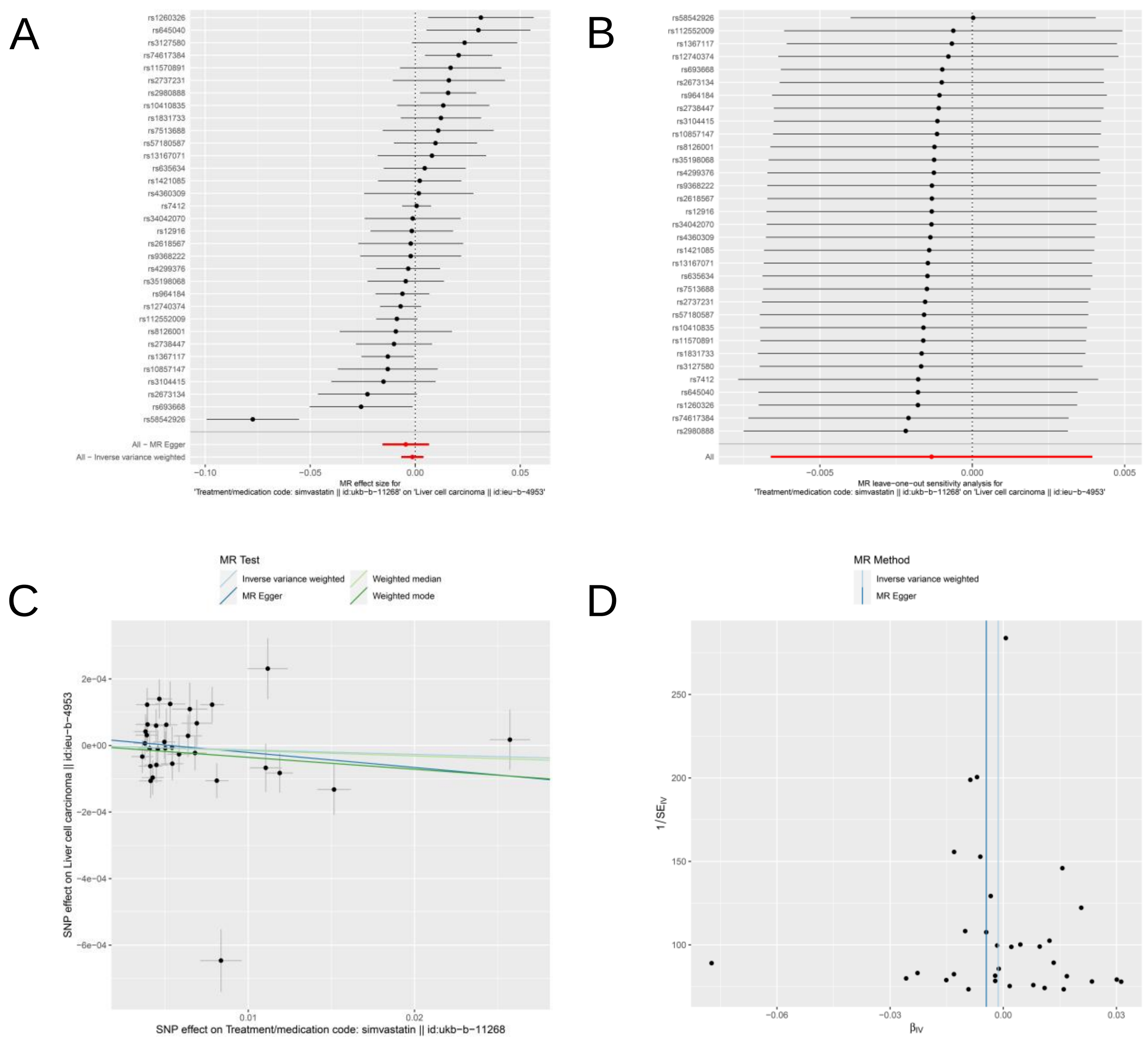

Cervical cancer

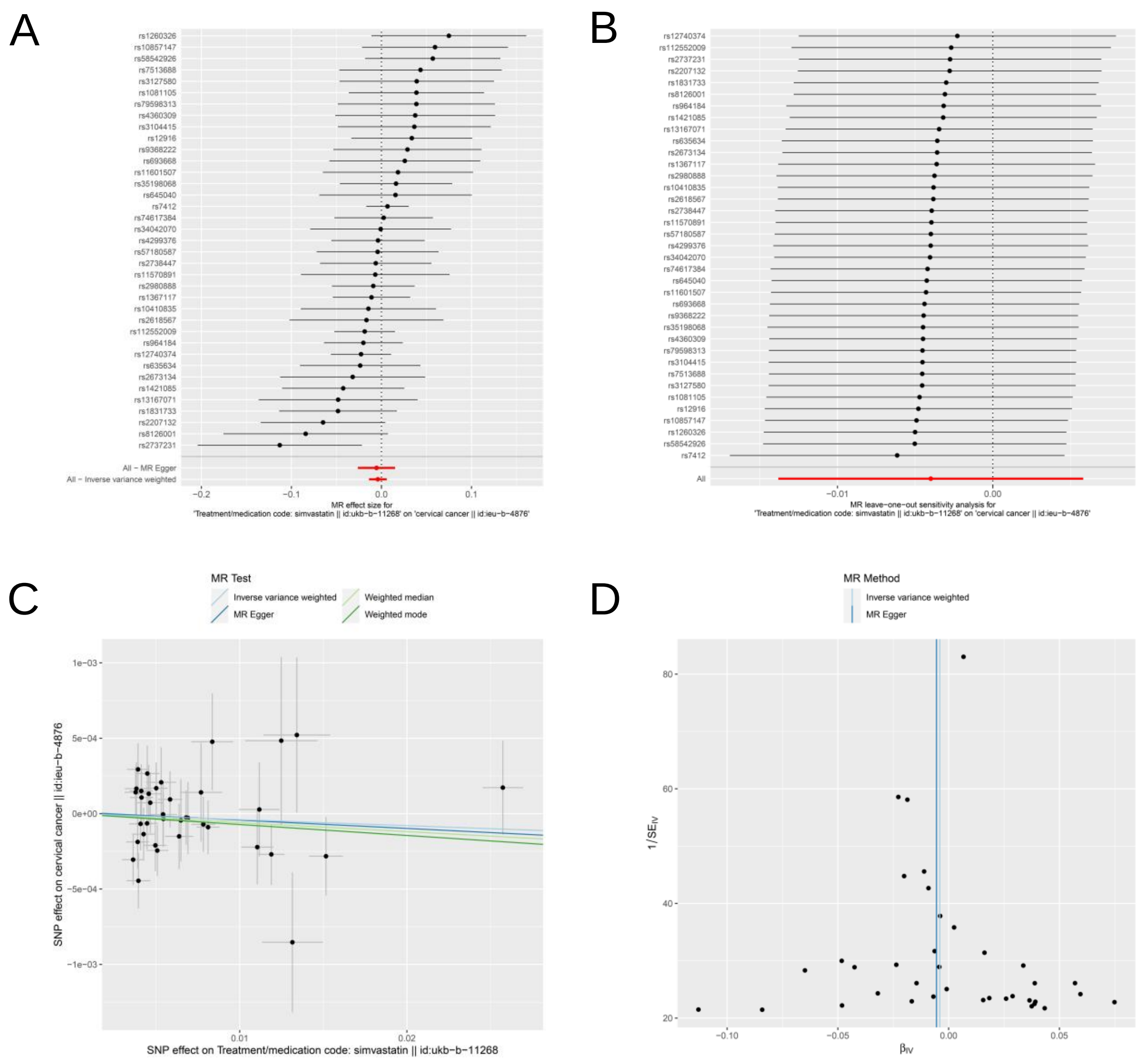

Colorectal cancer

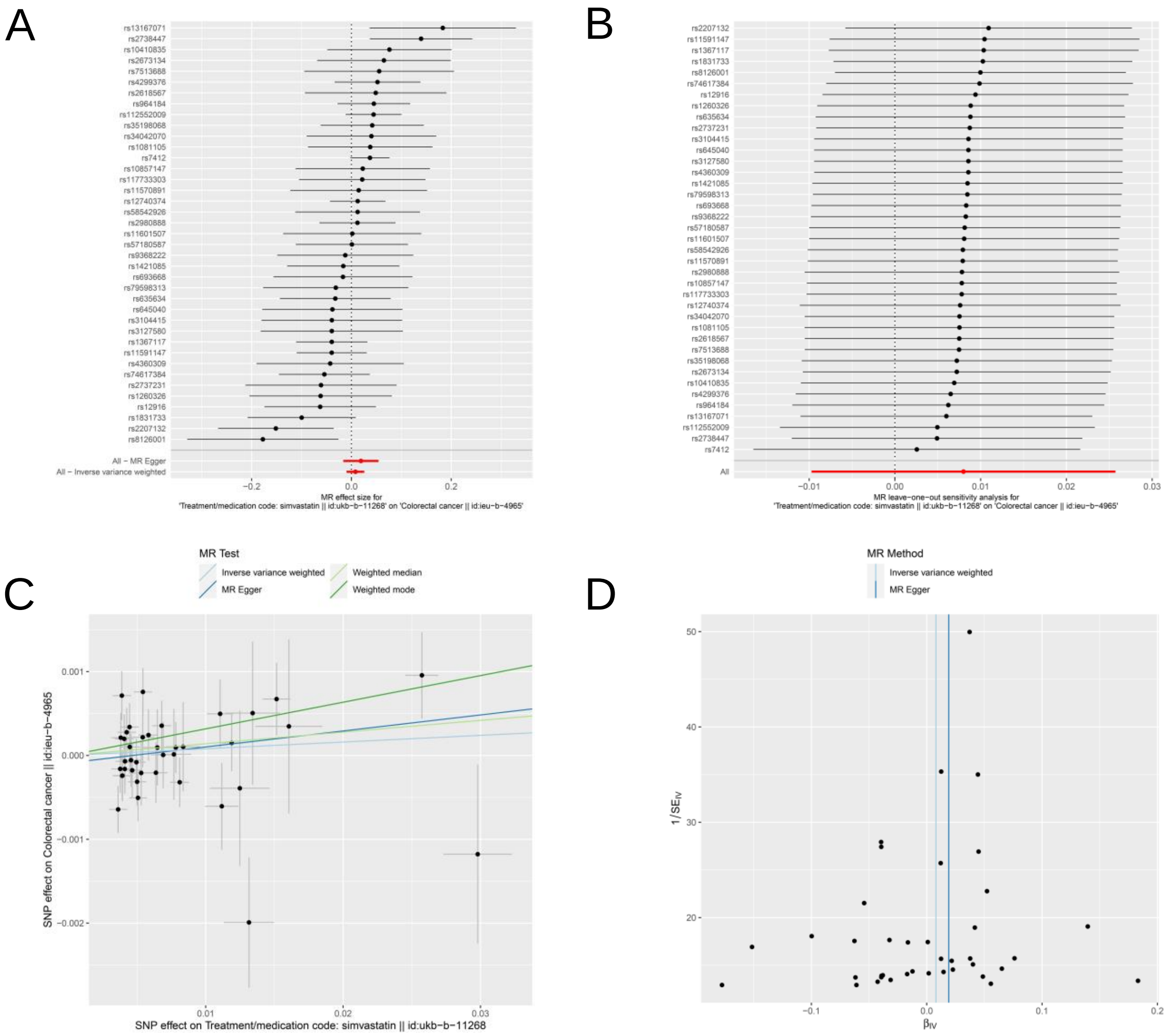

Ovarian cancer

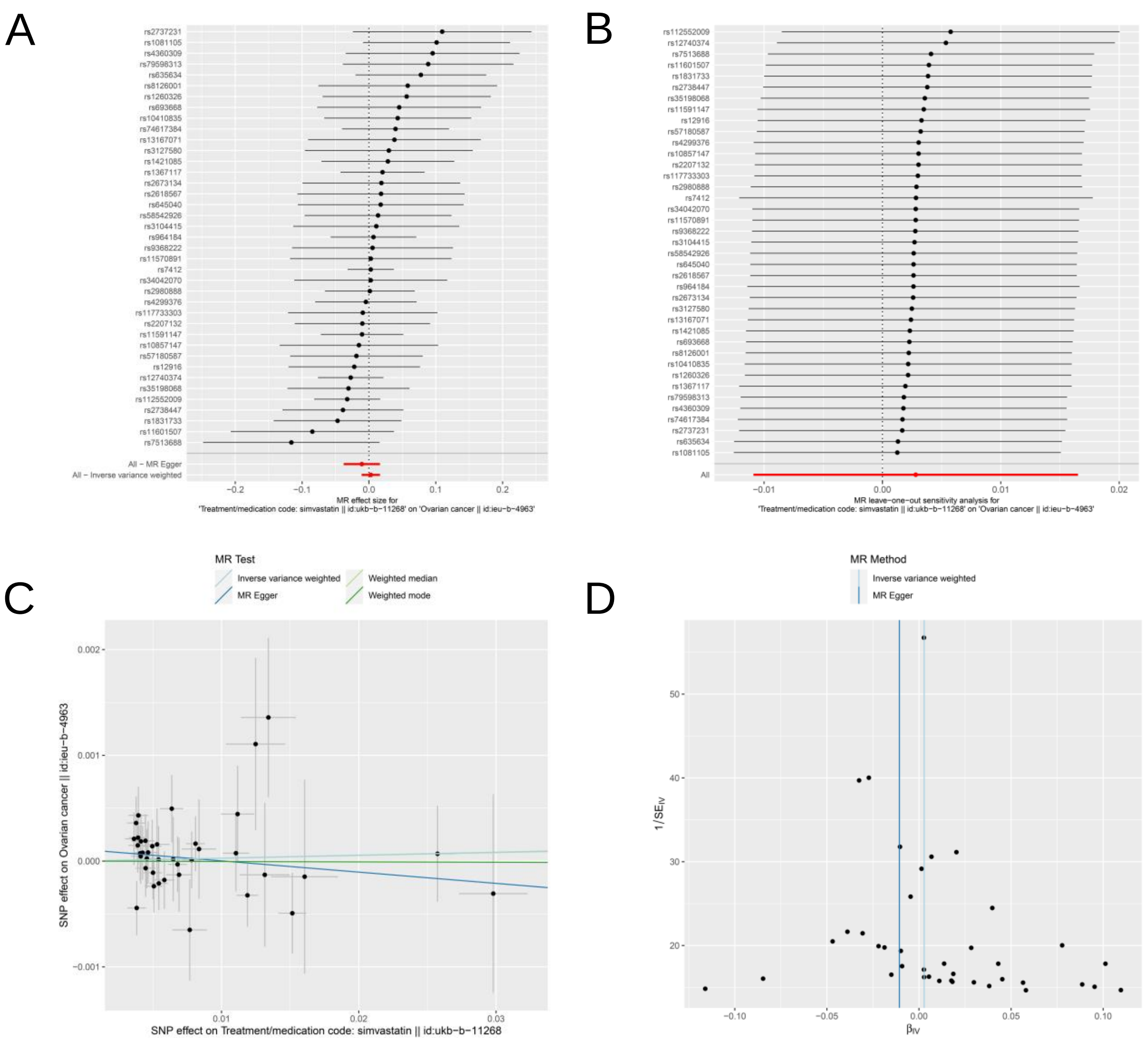

Non-melanoma

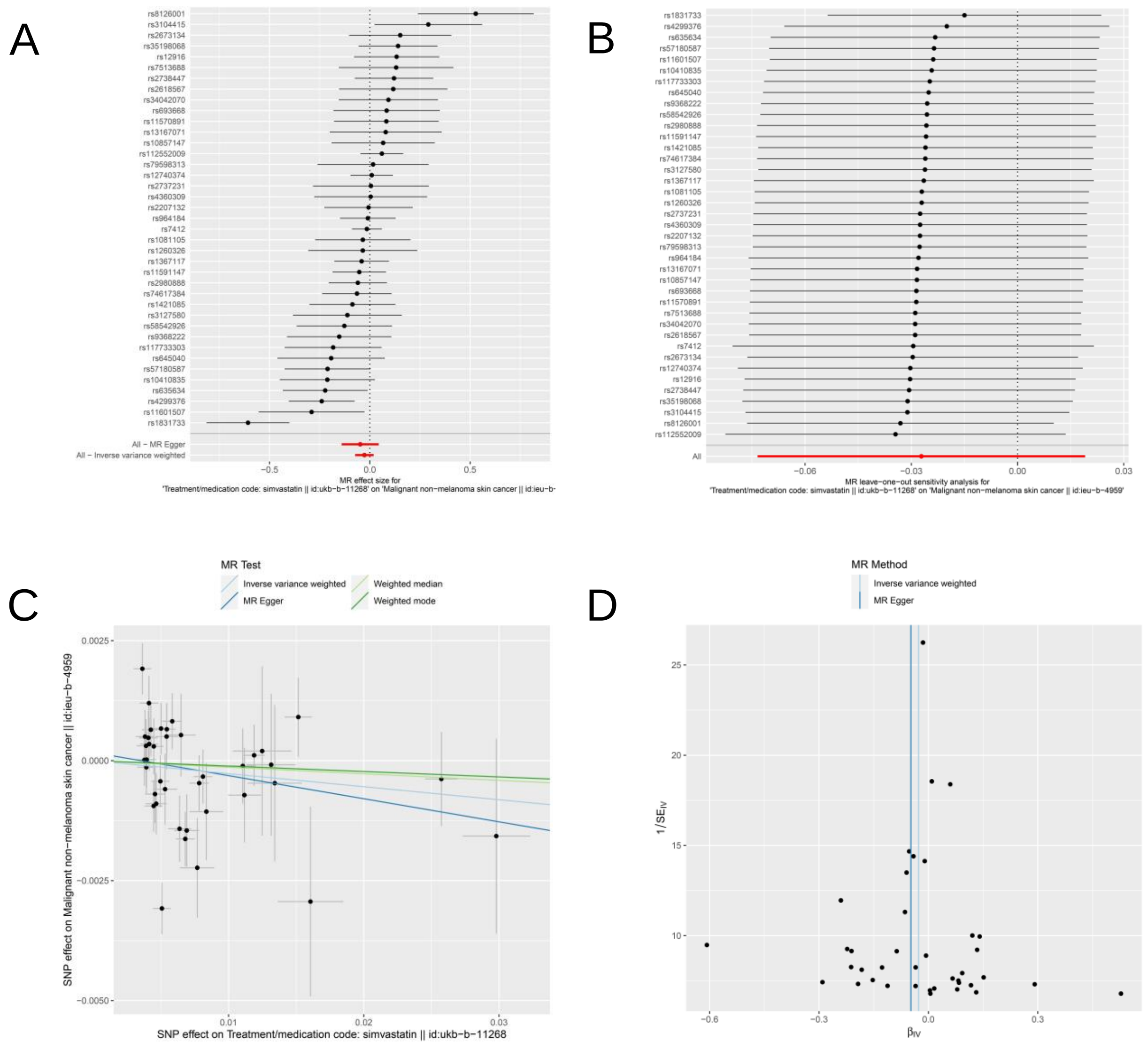

Melanoma

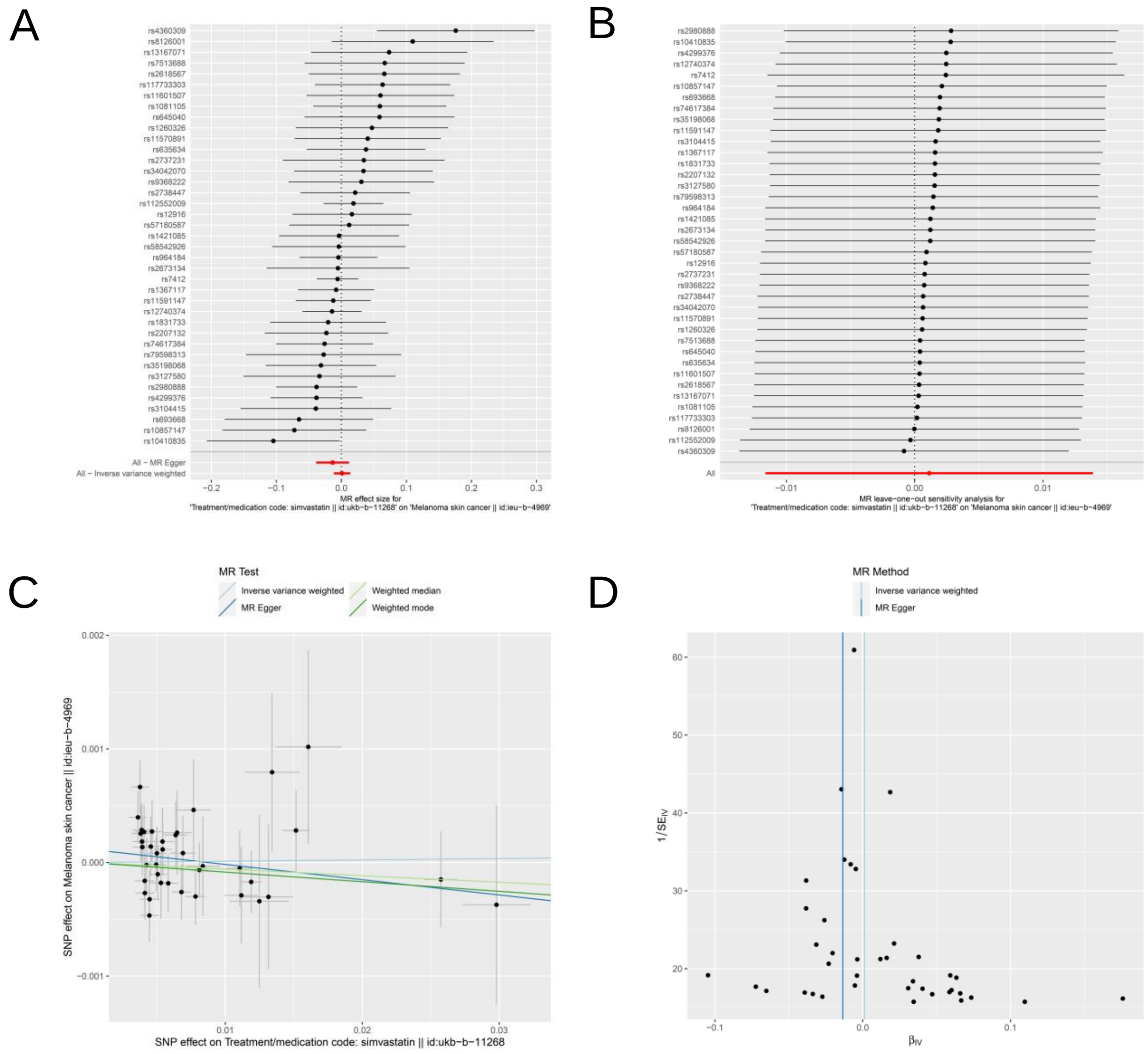

A
B
C
D
MR effect size for
'Treatment/medication code: simvastatin || id:ukb-b-11268' on 'Melanoma skin cancer || id:ieu-b-4969'
All – MR Egger
All – Inverse variance weighted
MR leave-one-out sensitivity analysis for
'Treatment/medication code: simvastatin || id:ukb-b-11268' on 'Melanoma skin cancer || id:ieu-b-4969'
All
MR Test
Inverse variance weighted
MR Egger
Weighted median
Weighted mode
SNP effect on Melanoma skin cancer || id:ieu-b-4969
SNP effect on Treatment/medication code: simvastatin || id:ukb-b-11268
MR Method
Inverse variance weighted
MR Egger
1/SE$_{IV}$
β$_{IV}$

Prostate cancer

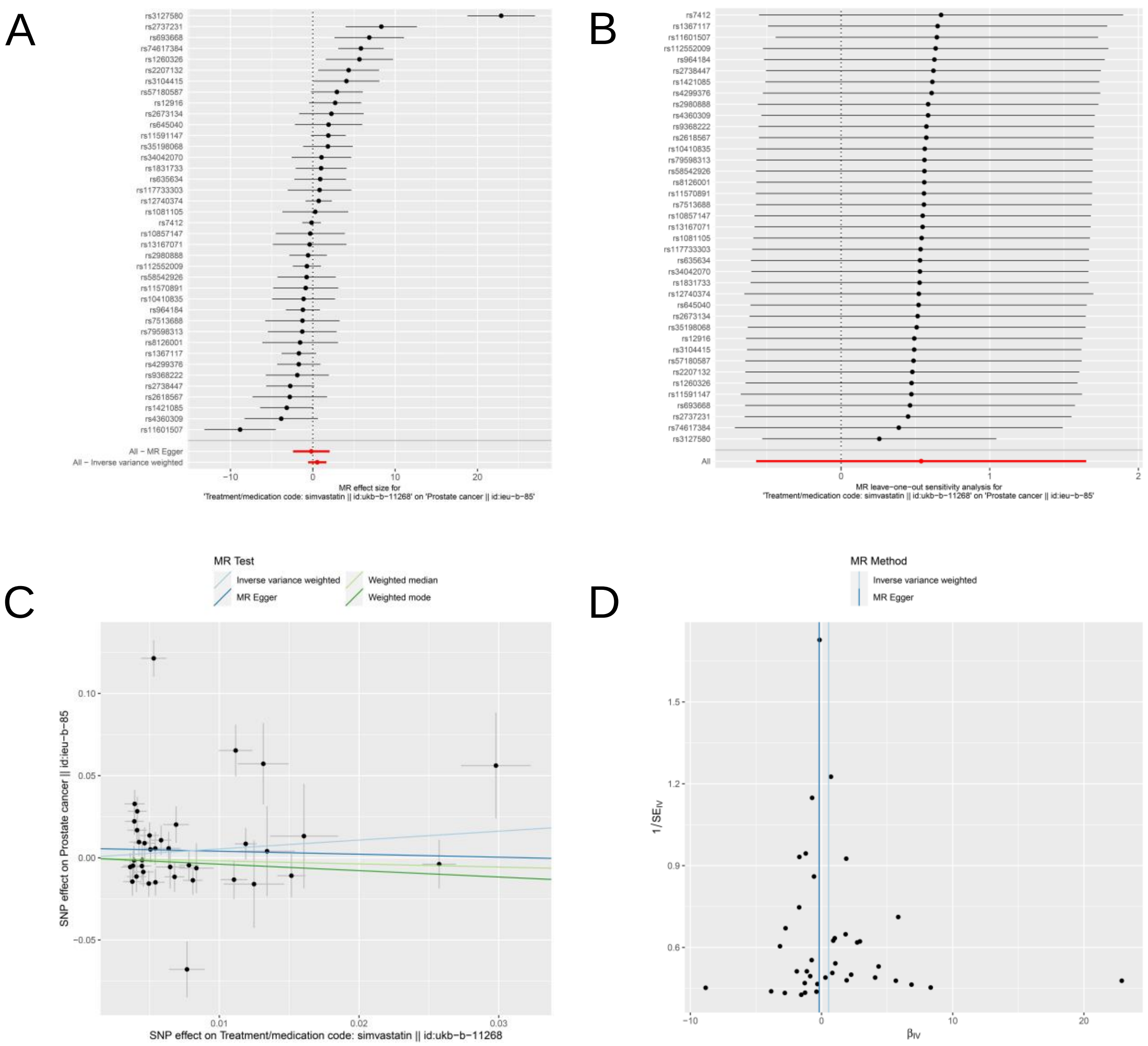

Breast cancer

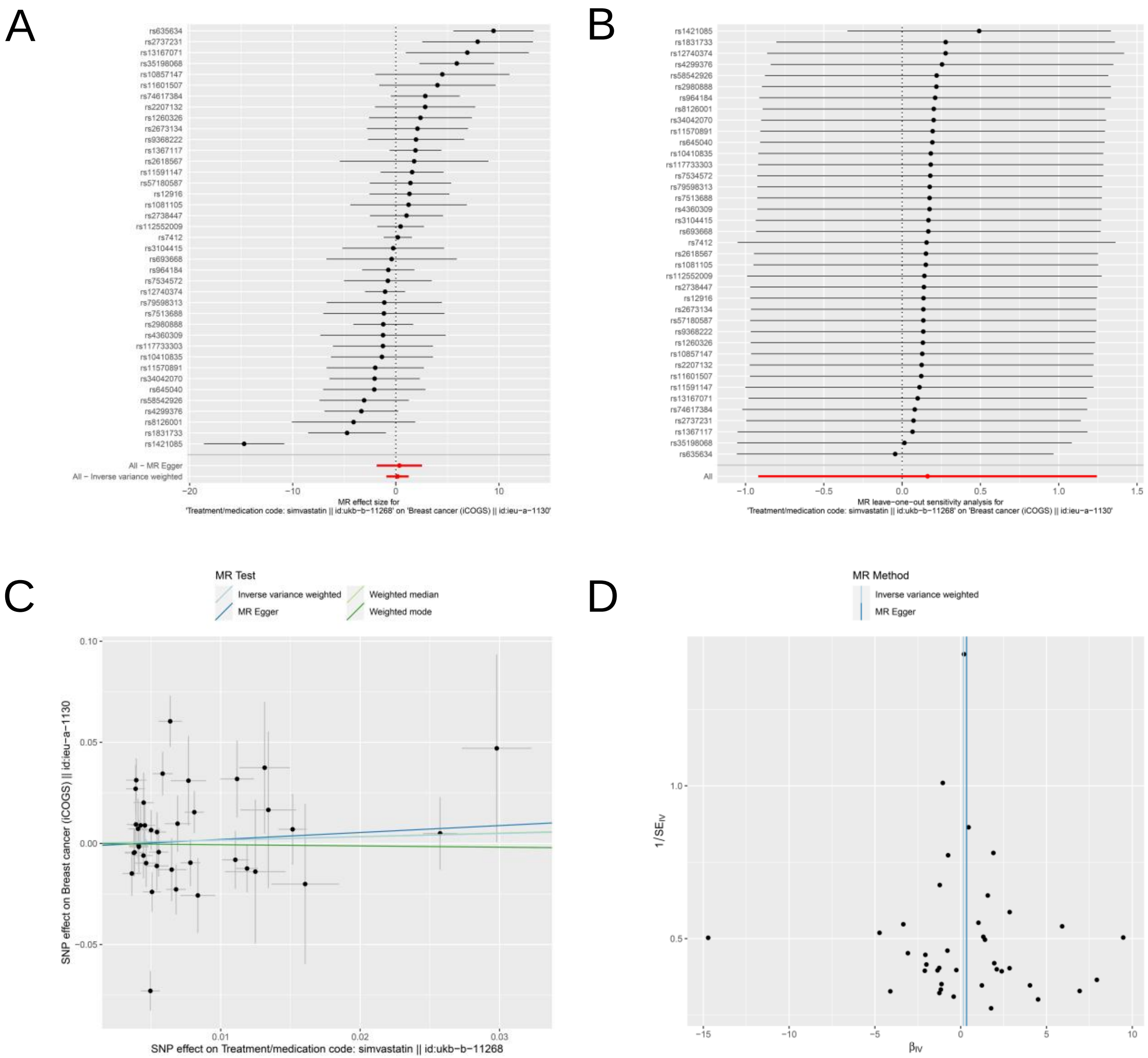

Esophagus cancer

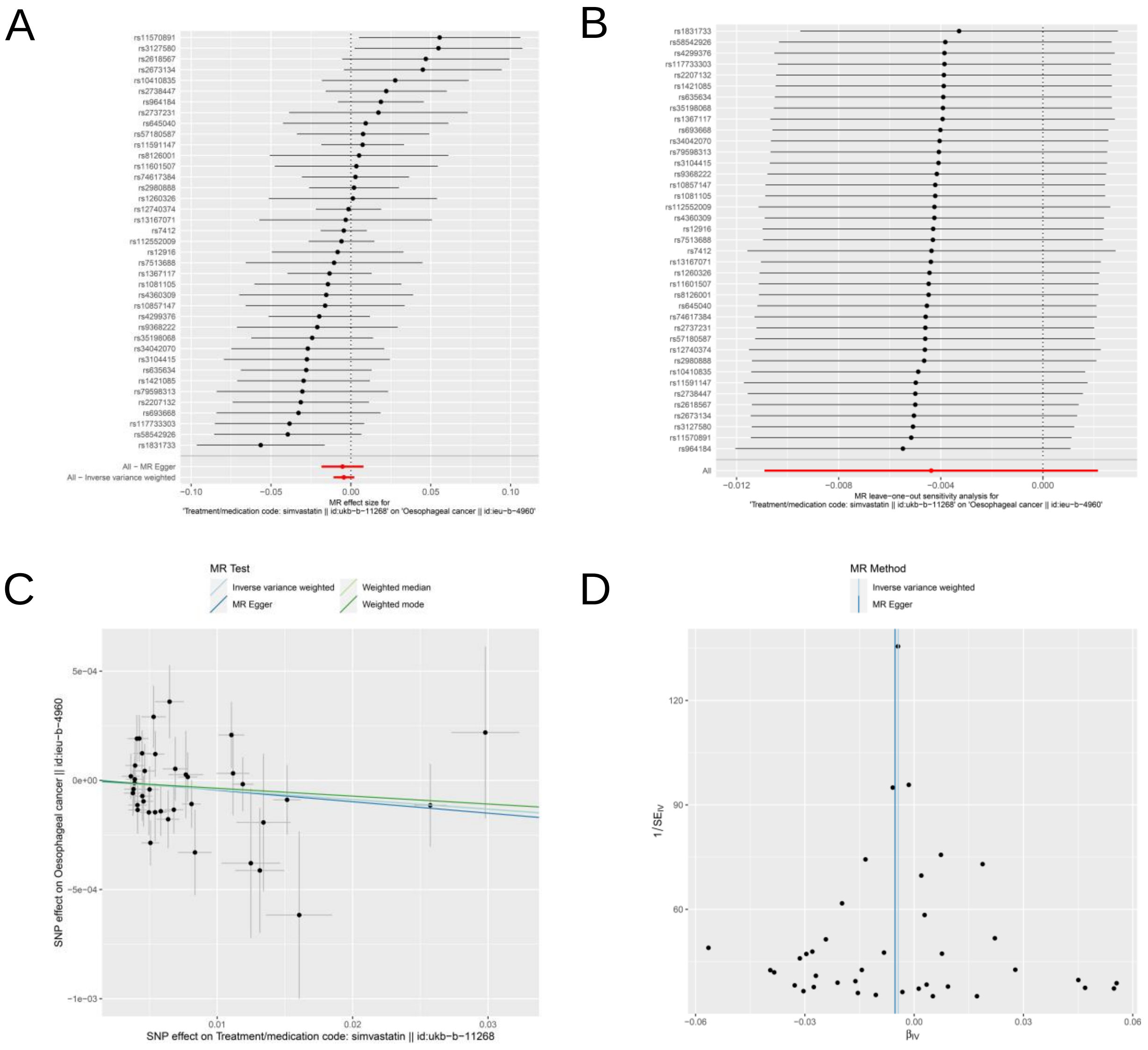

Head and neck cancer

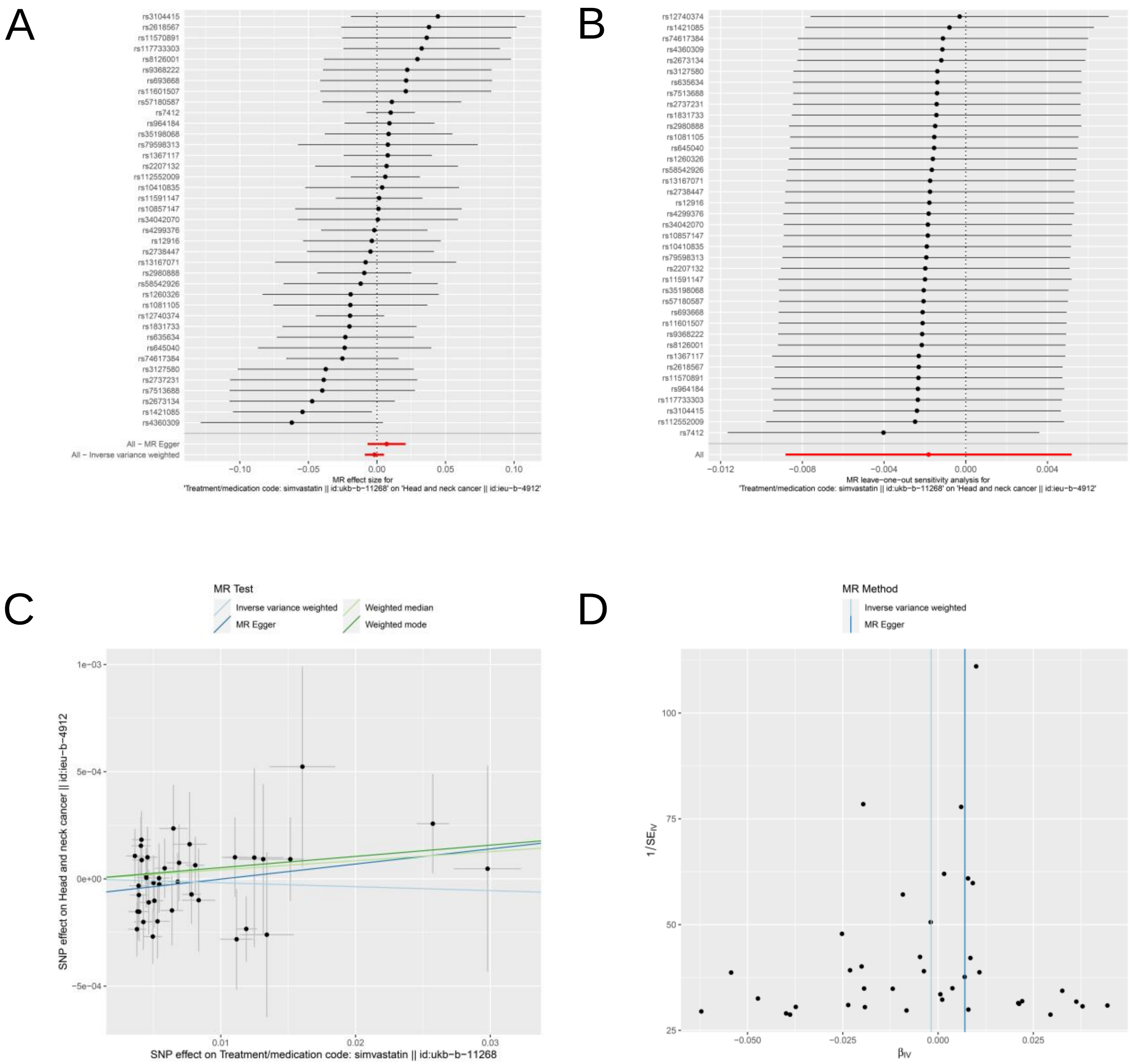

Figure S3. (A) The singly SNP estimated the causal effect of rosuvastatin use on pan-cancer risks; (B) the Leave-one-out analysis in estimating the causal effect of atorvastatin use on pan-cancer risks; (C) The four methods of MR test; (D) The scatter diagram for showing the tendency of dispersion in each cancer.

Bladder cancer

A

B

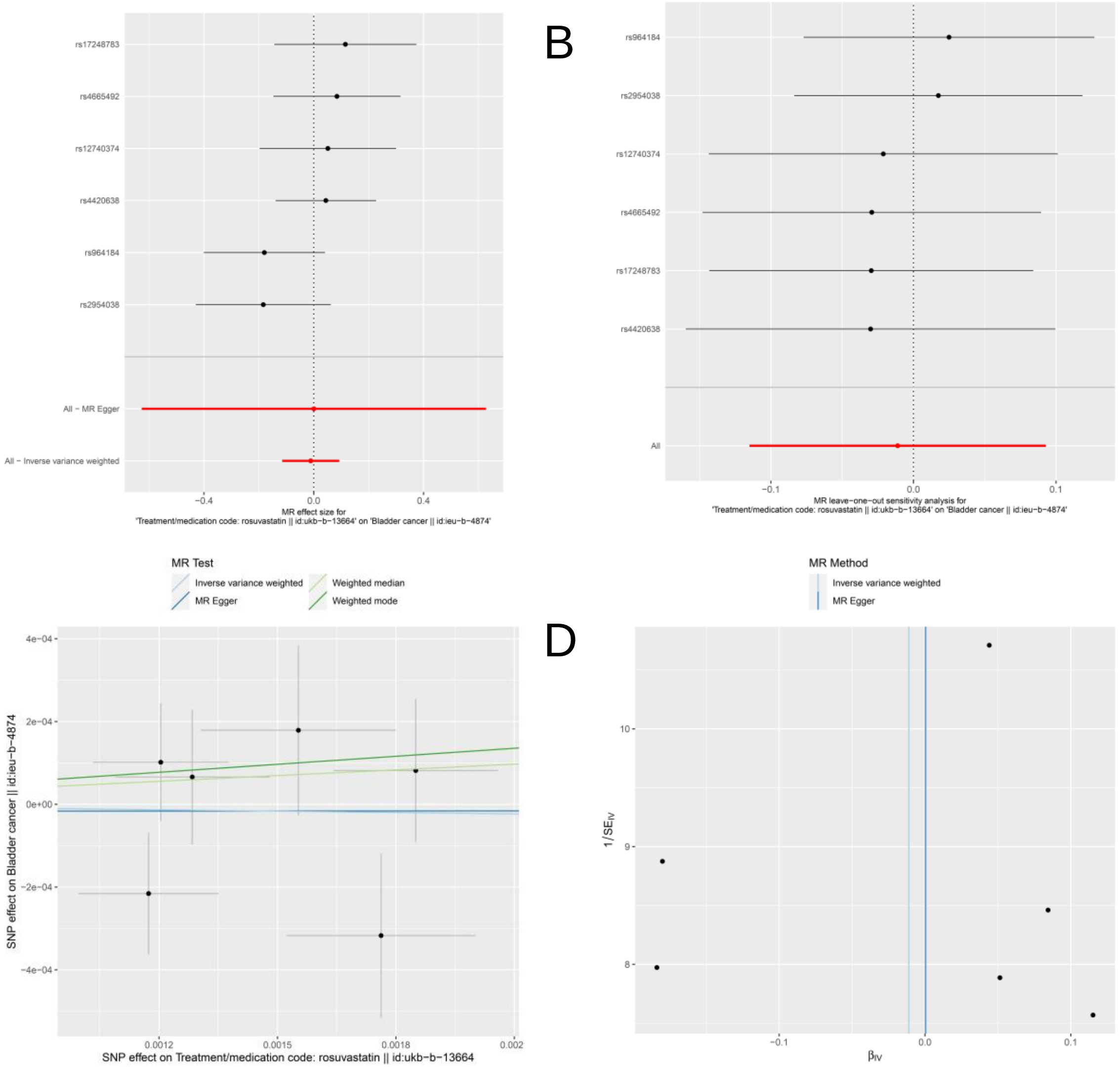

C

D

Lung cancer

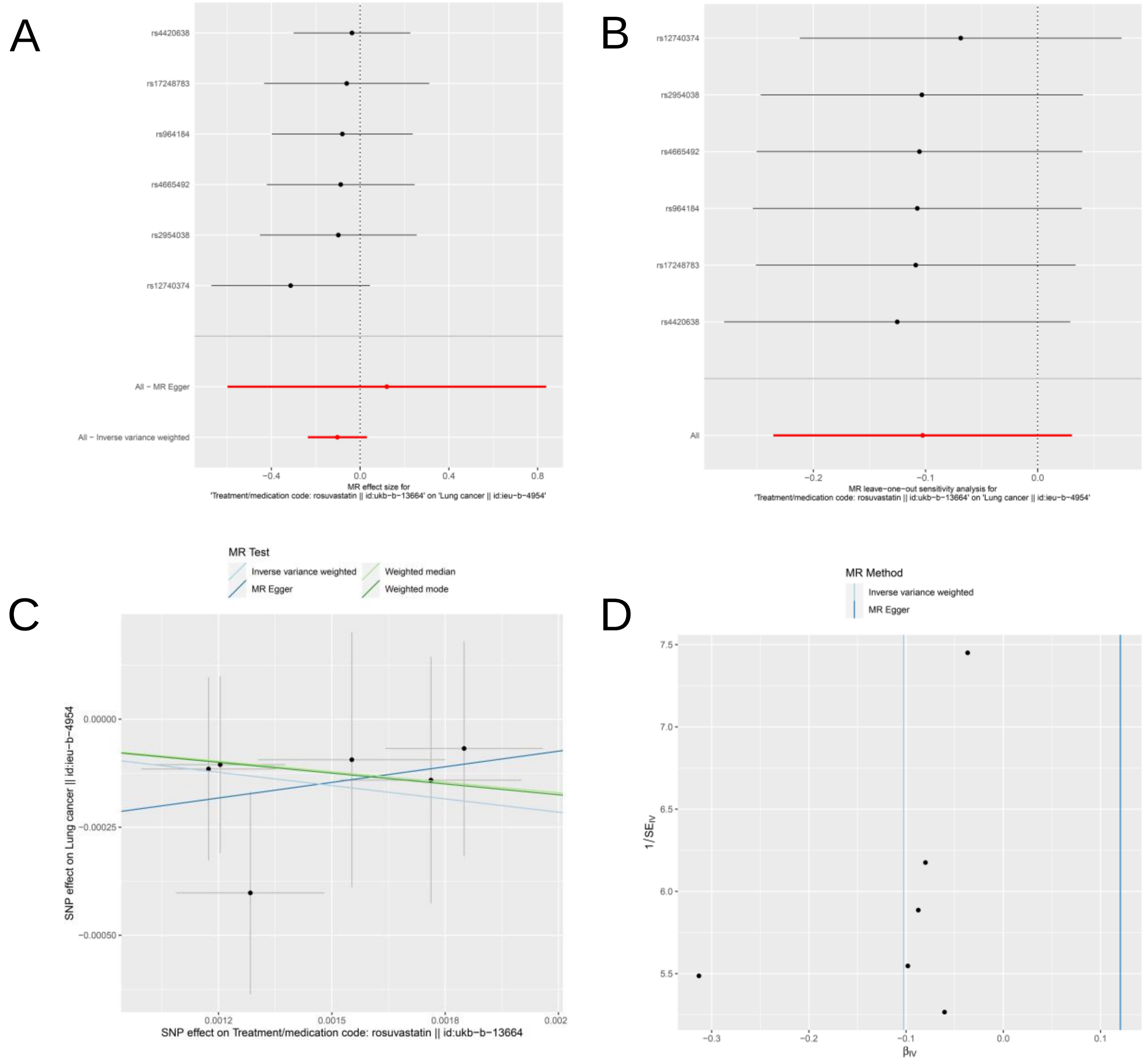
A
rs4420638
rs17248783
rs964184
rs4665492
rs2954038
rs12740374
All − MR Egger
All − Inverse variance weighted
−0.4
0.0
0.4
0.8
MR effect size for
'Treatment/medication code: rosuvastatin || id:ukb−b−13664' on 'Lung cancer || id:ieu−b−4954'
B
rs12740374
rs2954038
rs4665492
rs964184
rs17248783
rs4420638
All
−0.2
−0.1
0.0
MR leave−one−out sensitivity analysis for
'Treatment/medication code: rosuvastatin || id:ukb−b−13664' on 'Lung cancer || id:ieu−b−4954'
C
MR Test
Inverse variance weighted
MR Egger
Weighted median
Weighted mode
0.00000
−0.00025
−0.00050
SNP effect on Lung cancer || id:ieu−b−4954
0.0012
0.0015
0.0018
0.002
SNP effect on Treatment/medication code: rosuvastatin || id:ukb−b−13664
D
MR Method
Inverse variance weighted
MR Egger
7.5
7.0
6.5
6.0
5.5
$1/SE_{IV}$
−0.3
−0.2
−0.1
0.0
0.1
β_{IV}

Bile ductal cancer

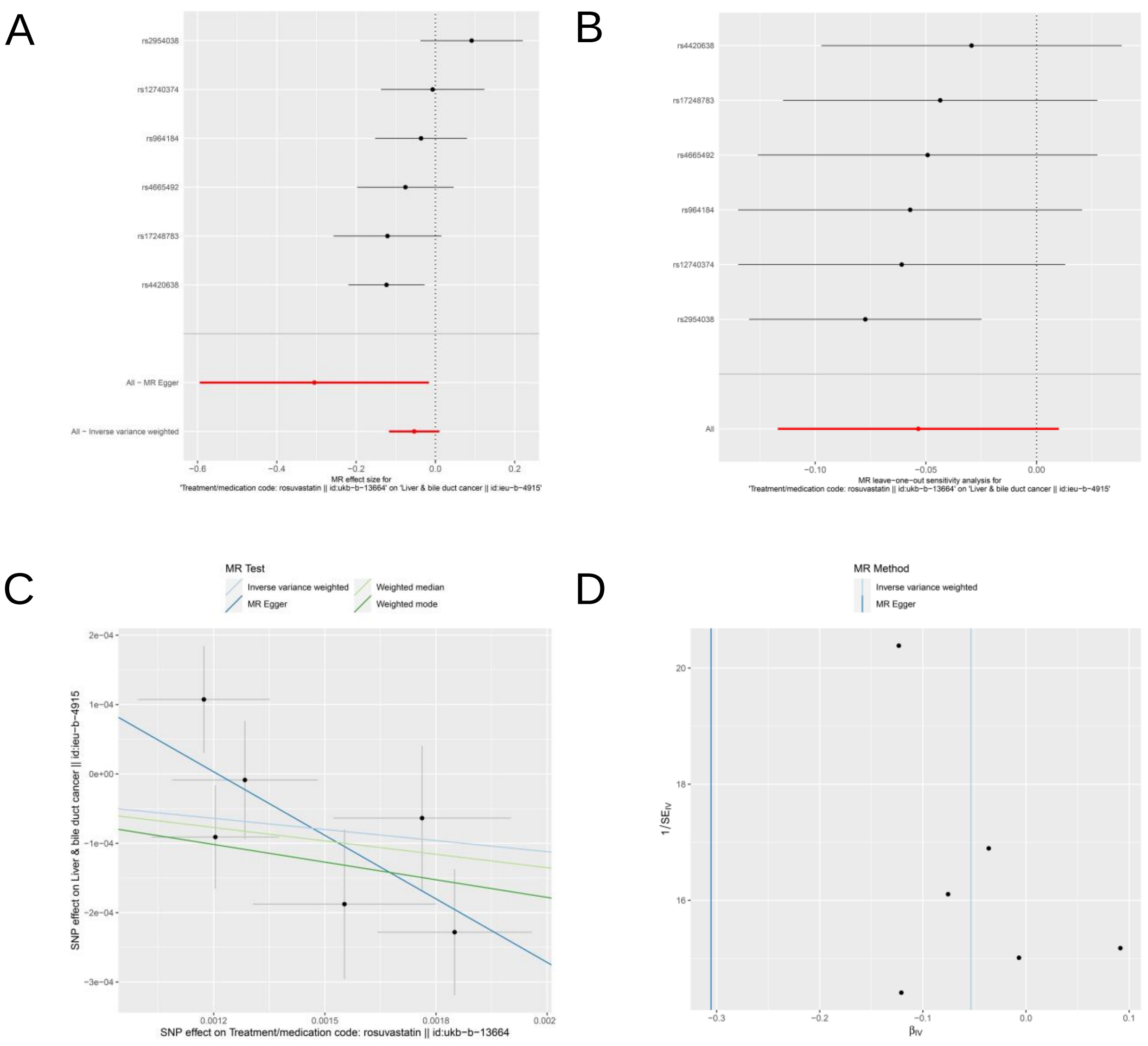

Liver cancer

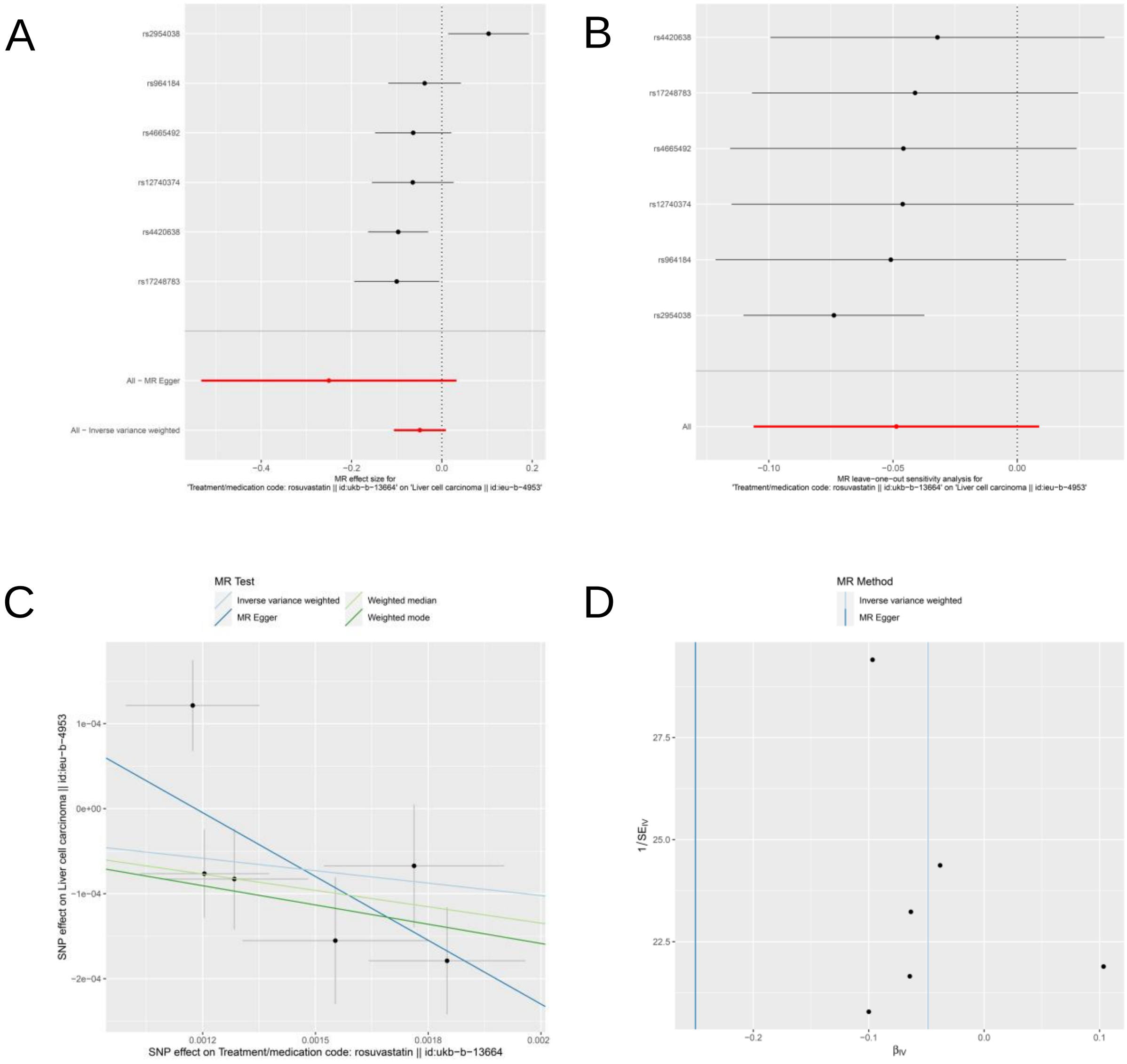

Cervical cancer

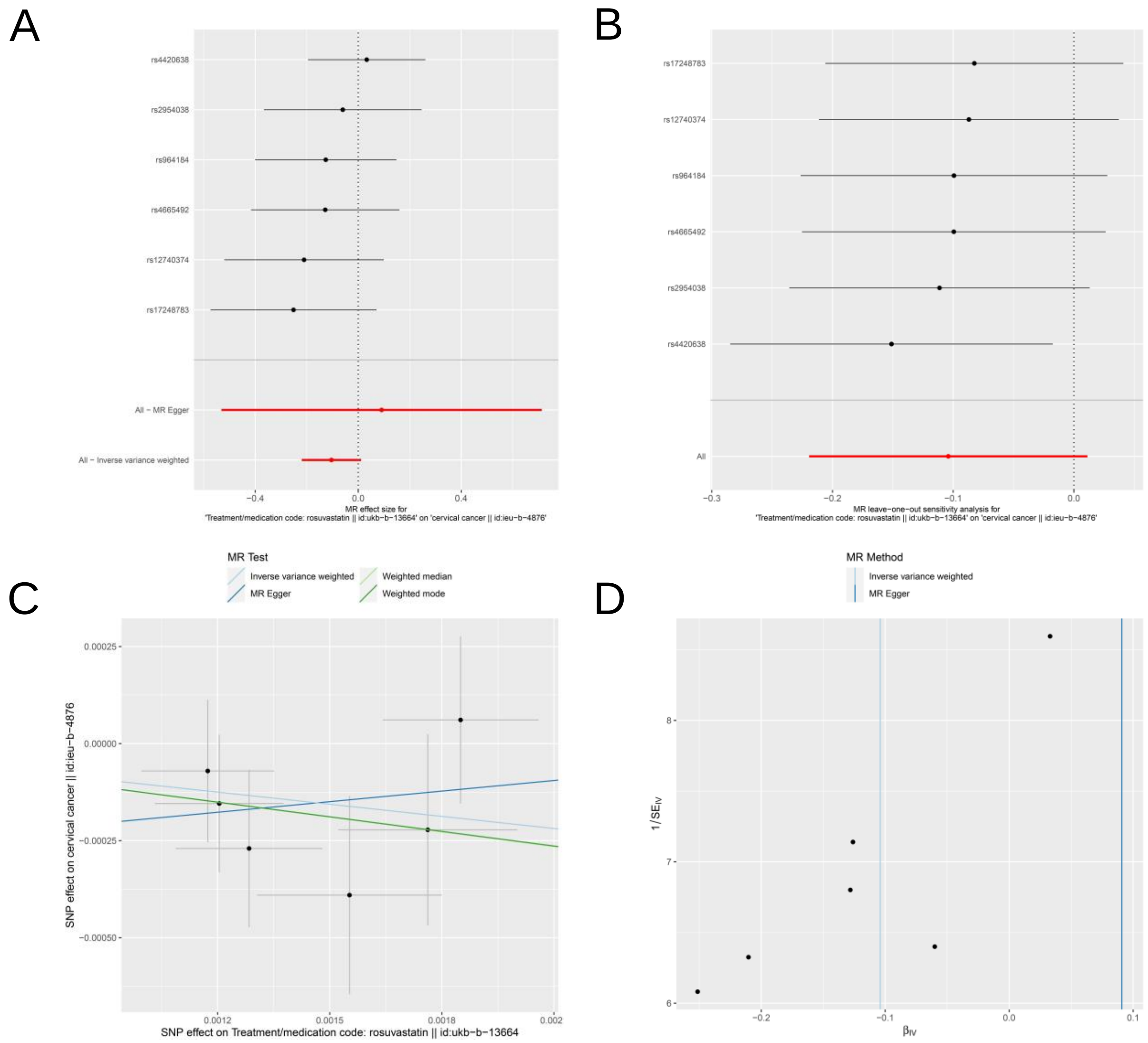

Colorectal cancer

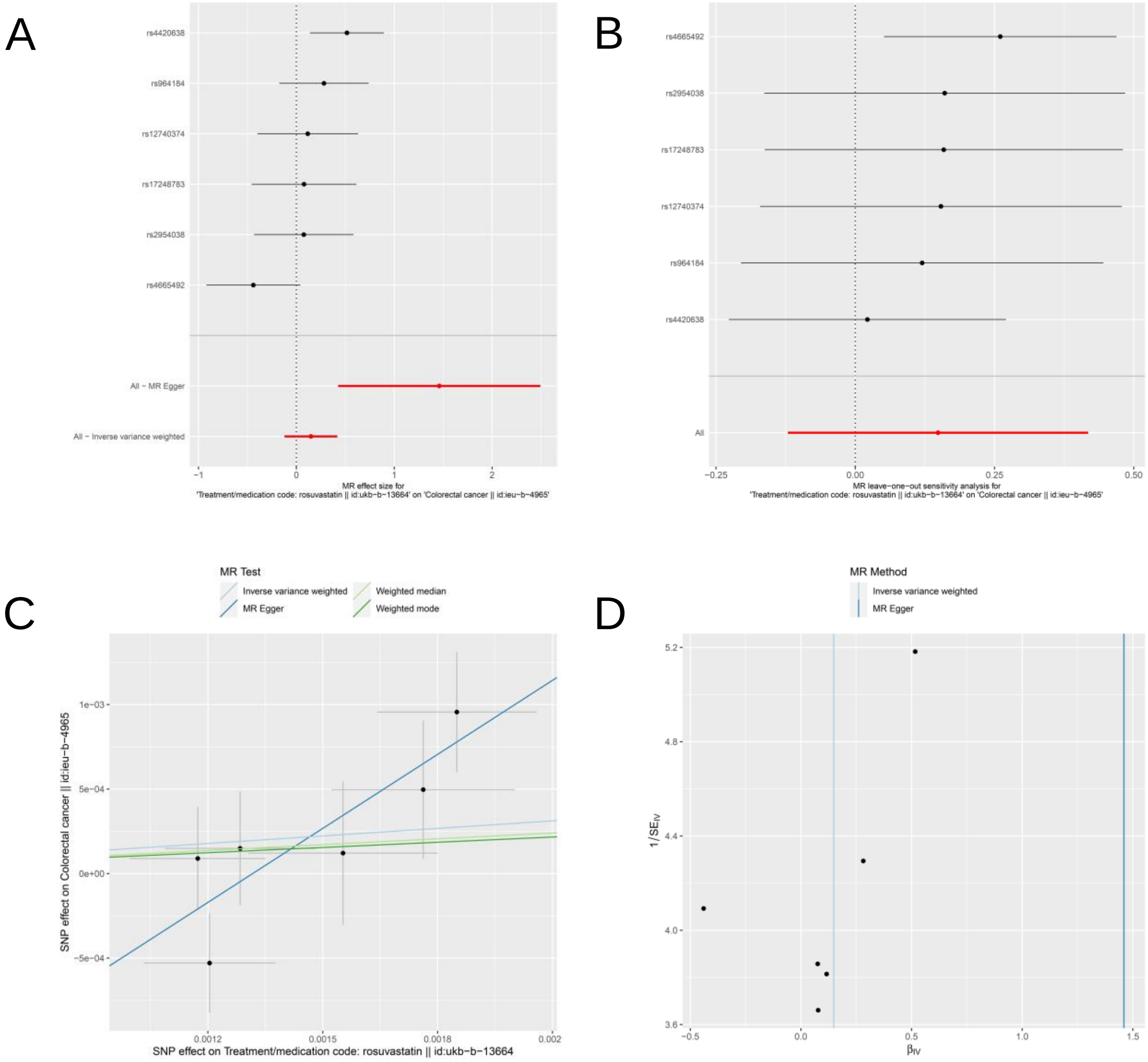

Ovarian cancer

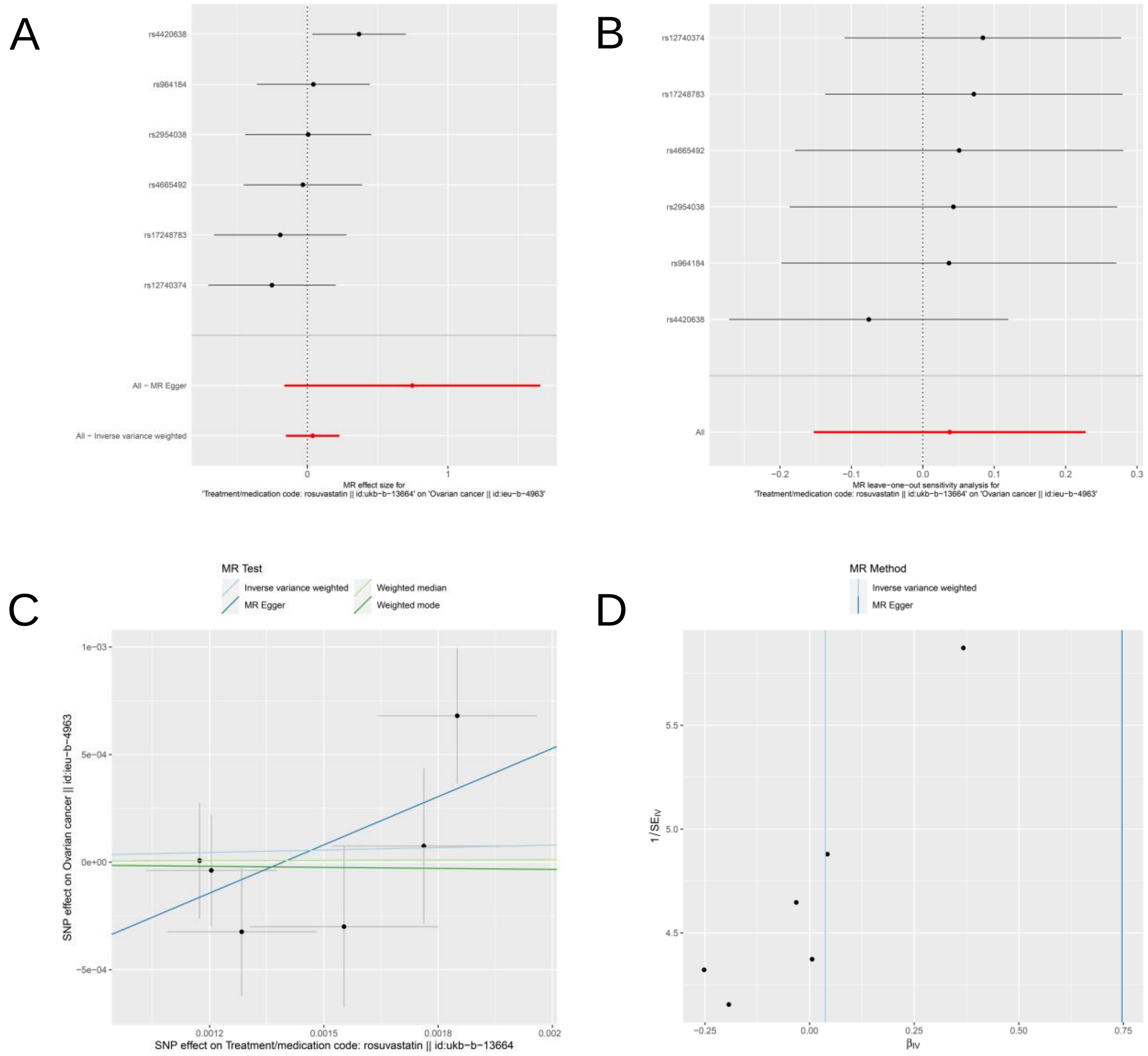

Non-melanoma

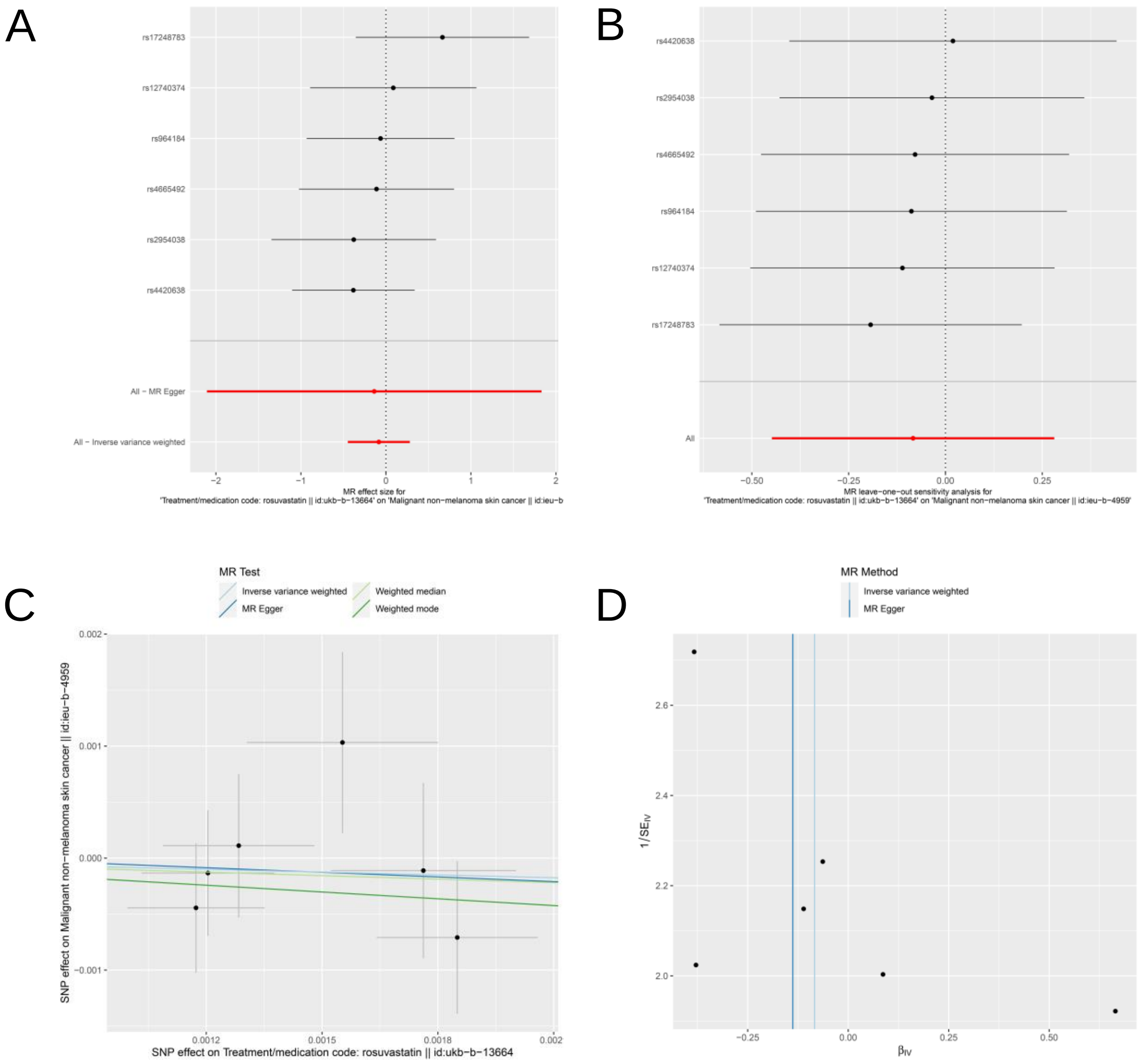

Melanoma

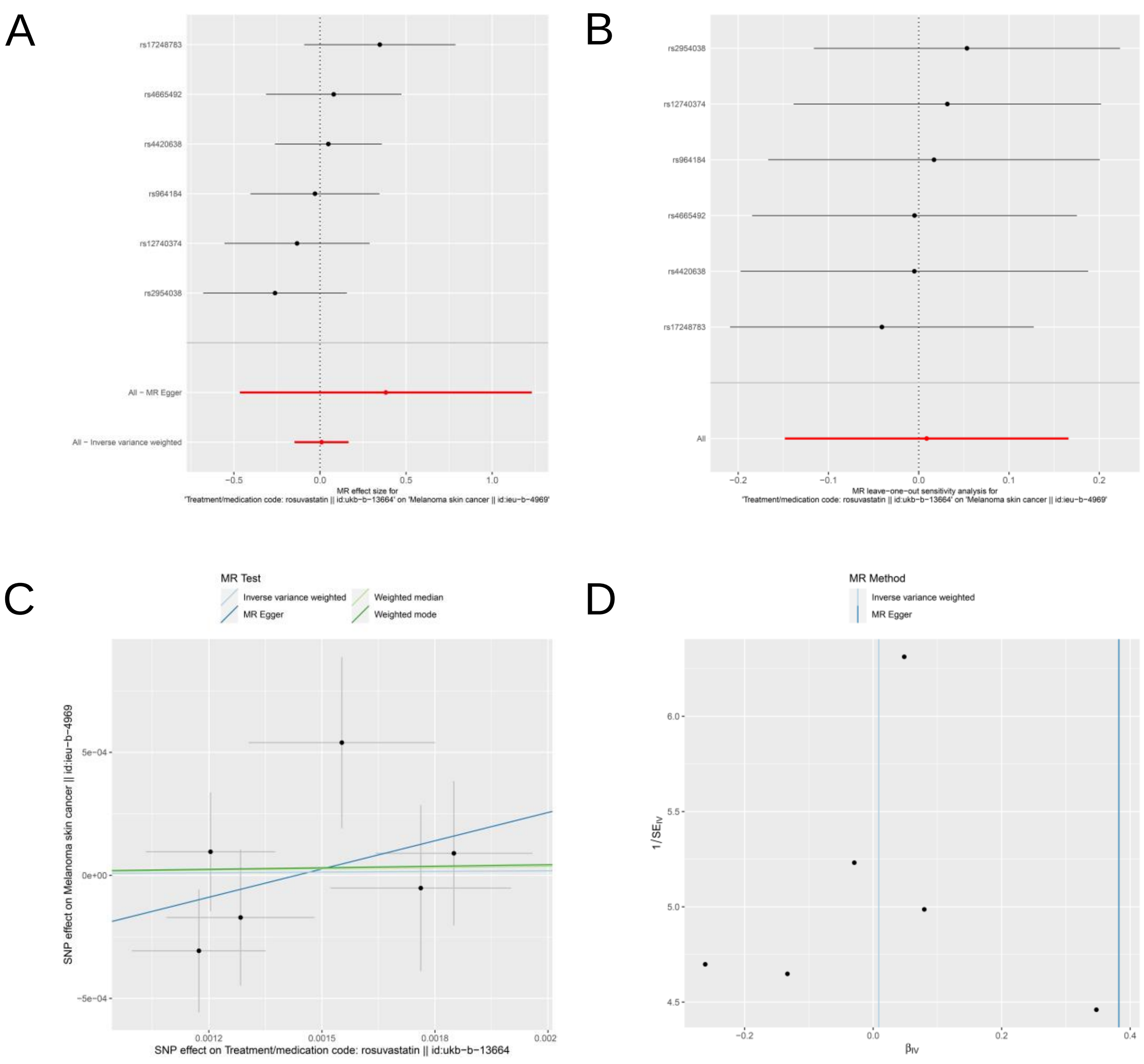
A
B
C
D
rs17248783
rs4665492
rs4420638
rs964184
rs12740374
rs2954038
All − MR Egger
All − Inverse variance weighted
MR effect size for
'Treatment/medication code: rosuvastatin || id:ukb−b−13664' on 'Melanoma skin cancer || id:ieu−b−4969'
All
MR leave−one−out sensitivity analysis for
'Treatment/medication code: rosuvastatin || id:ukb−b−13664' on 'Melanoma skin cancer || id:ieu−b−4969'
MR Test
Inverse variance weighted
MR Egger
Weighted median
Weighted mode
SNP effect on Melanoma skin cancer || id:ieu−b−4969
SNP effect on Treatment/medication code: rosuvastatin || id:ukb−b−13664
MR Method
Inverse variance weighted
MR Egger
1/SE
β

Esophageal cancer

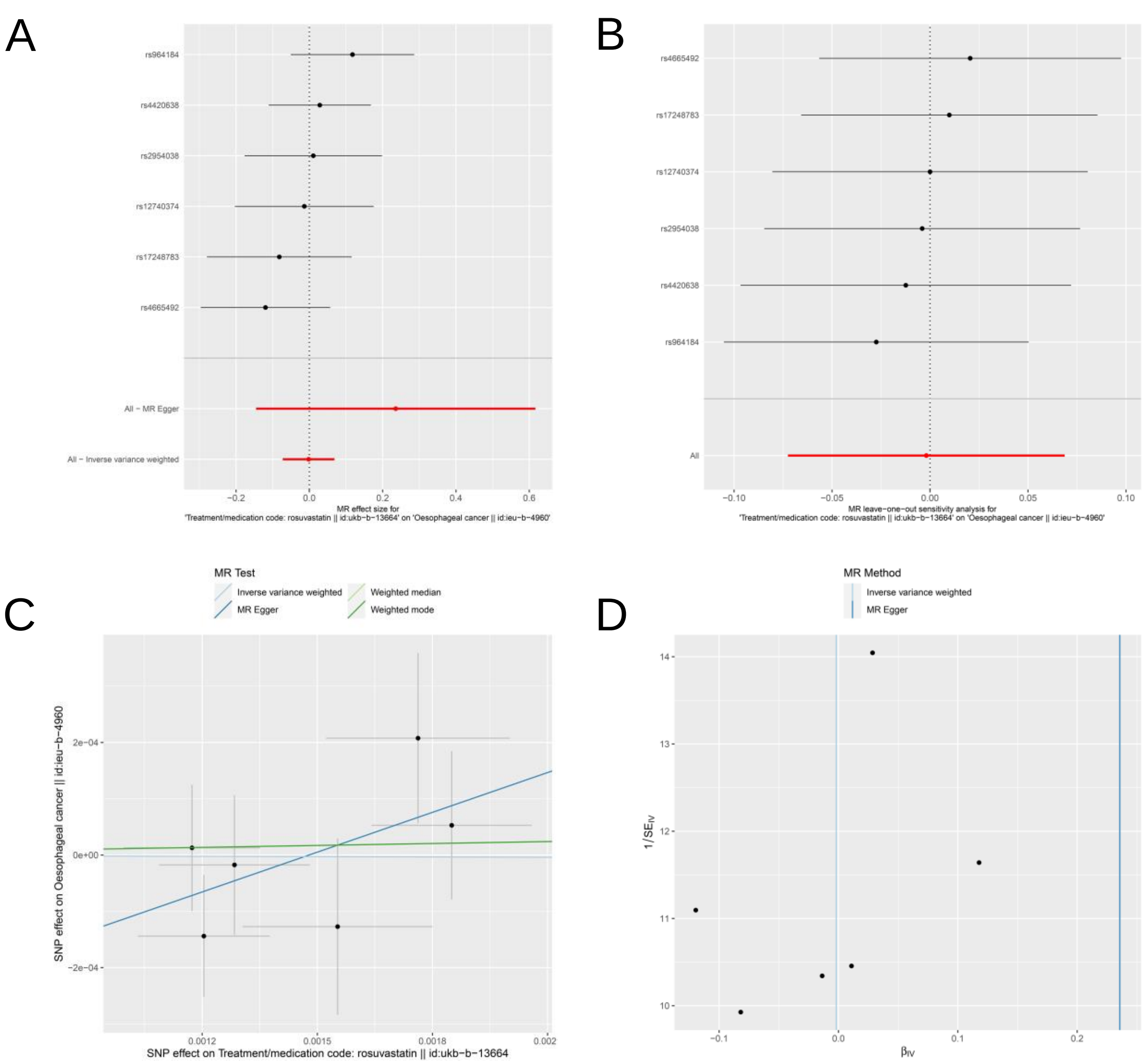

Head and neck cancer

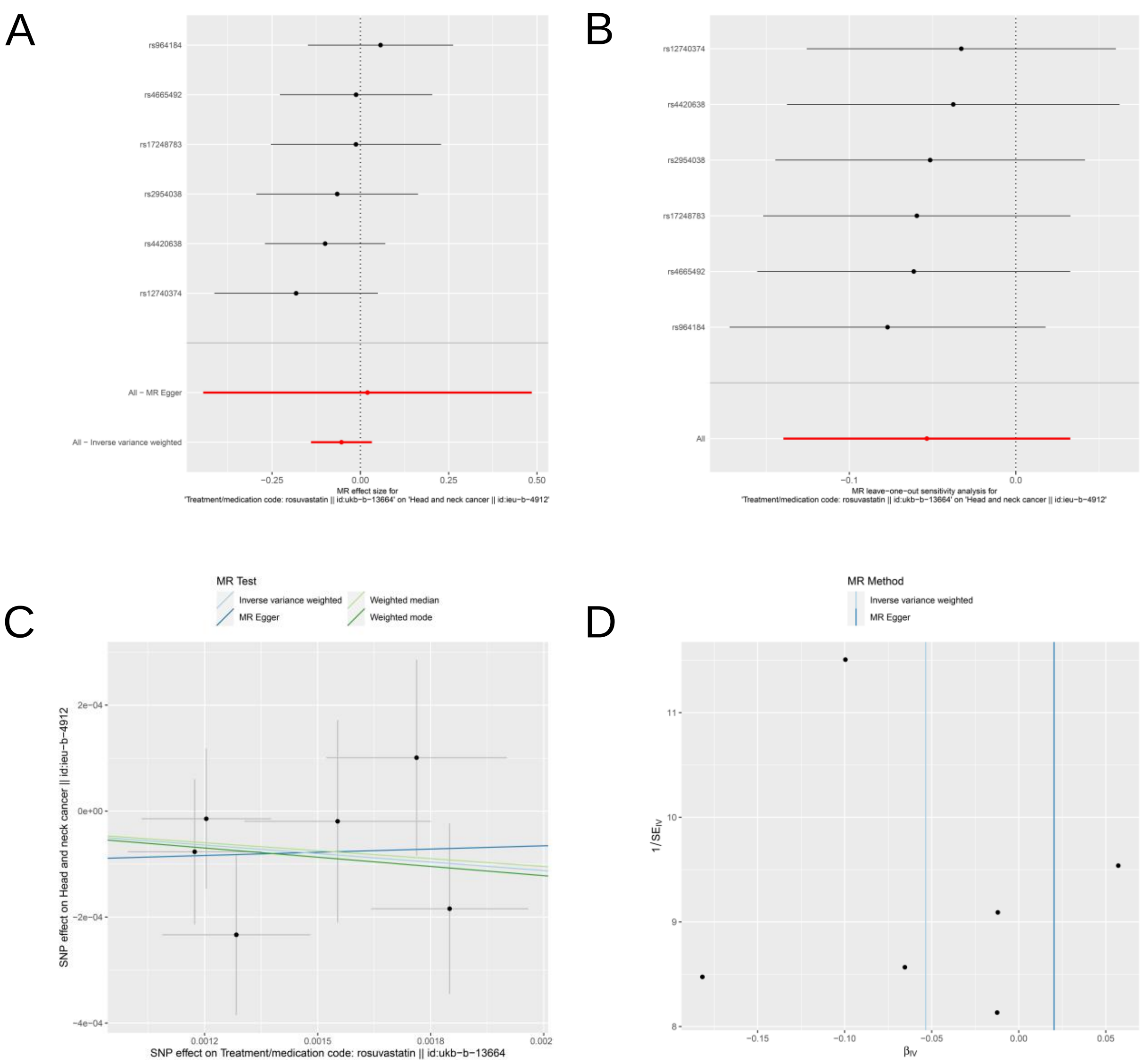

Supplement: Supplementary file 1 [file DataSheet2.PDF]
