# Supplementary material for: Assessing the role of lipid-lowering therapy on multi-cancer prevention: A mendelian randomization study
Source: Front Pharmacol. 2023 Apr 19;14:1109580. doi: 10.3389/fphar.2023.1109580 (PMC10154601; doi:10.3389/fphar.2023.1109580)
Supplement: Supplementary file 2 [file DataSheet1.ZIP › Table S4 Atorvastatin.docx]

**Table S4.** Two sample MR analysis results of atorvastatin use and pan-cancer.

| **Outcome** | **MR Method** | **No. SNPs** | **β** | **SE** | **OR (95%CI)** | ***P*** |
| --- | --- | --- | --- | --- | --- | --- |
| **Bladder** | MR Egger | 22 | 0.018 | 0.026 | 1.018 (0.969-1.071) | 0.482 |
|  | Weighted median | 22 | 0.003 | 0.014 | 1.003(0.975-1.031) | 0.858 |
|  | Weighted mode | 22 | 0.005 | 0.017 | 1.005(0.972-1.040) | 0.776 |
| **Lung** | MR Egger | 22 | -0.014 | 0.044 | 0.986(0.904-1.075) | 0.756 |
|  | Weighted median | 22 | -0.010 | 0.020 | 0.990(0.951-1.030) | 0.608 |
|  | Weighted mode | 22 | -0.013 | 0.024 | 0.987(0.942-1.036) | 0.610 |
| **Bile duct** | MR Egger | 21 | -0.015 | 0.018 | 0.985(0.952-1.020) | 0.405 |
|  | Weighted median | 21 | -0.006 | 0.008 | 0.994(0.979-1.009) | 0.427 |
|  | Weighted mode | 21 | -0.009 | 0.013 | 0.991(0.966-1.018) | 0.519 |
| **Liver cell** | MR Egger | 21 | -0.014 | 0.017 | 0.986(0.954-1.019) | 0.405 |
|  | Weighted median | 21 | -0.011 | 0.006 | 0.989(0.978-0.999) | **0.049** |
|  | Weighted mode | 21 | -0.016 | 0.007 | 0.984(0.970-0.998) | **0.040** |
| **Cervical** | MR Egger | 22 | -0.028 | 0.028 | 0.972(0.920-1.027) | 0.325 |
|  | Weighted median | 22 | -0.007 | 0.017 | 0.993(0.962-1.026) | 0.688 |
|  | Weighted mode | 22 | -0.017 | 0.021 | 0.983(0.944-1.025) | 0.430 |
| **Colorectal** | MR Egger | 22 | 0.024 | 0.059 | 1.025(0.913-1.150) | 0.682 |
|  | Weighted median | 22 | 0.082 | 0.029 | 1.086(1.025-1.150) | **0.005** |
|  | Weighted mode | 22 | 0.097 | 0.046 | 1.101(1.006-1.205) | **0.048** |
| **Ovarian** | MR Egger | 22 | -0.011 | 0.041 | 0.989(0.913-1.071) | 0.785 |
|  | Weighted median | 22 | 0.011 | 0.024 | 1.011(0.964-1.061) | 0.644 |
|  | Weighted mode | 22 | 0.015 | 0.037 | 1.015(0.944-1.091) | 0.693 |
| **Non-melanoma** | MR Egger | 22 | -0.009 | 0.101 | 0.991(0.813-1.208) | 0.931 |
|  | Weighted median | 22 | -0.029 | 0.052 | 0.972(0.877-1.077) | 0.583 |
|  | Weighted mode | 22 | -0.032 | 0.071 | 0.968(0.843-1.112) | 0.654 |
| **Melanoma** | MR Egger | 22 | -0.015 | 0.038 | 0.985(0.914-1.062) | 0.700 |
|  | Weighted median | 22 | 0.009 | 0.021 | 1.009(0.969-1.051) | 0.662 |
|  | Weighted mode | 22 | 0.002 | 0.028 | 1.002(0.949-1.059) | 0.934 |
| **Prostate** | MR Egger | 22 | -0.028 | 2.469 | 0.973(0.008-122.884) | 0.991 |
|  | Weighted median | 22 | -0.084 | 0.977 | 0.919 (0.135-6.238) | 0.931 |
|  | Weighted mode | 22 | -0.202 | 1.648 | 0.817 (0.032-20.648) | 0.904 |
| **Breast** | MR Egger | 21 | -2.746 | 2.784 | 0.064(0.000-15.030) | 0.336 |
|  | Weighted median | 21 | -0.321 | 1.012 | 0.725(0.100-5.269) | 0.751 |
|  | Weighted mode | 21 | -0.839 | 1.501 | 0.432(0.023-8.195) | 0.582 |
| **Oesophagus** | MR Egger | 22 | -0.001 | 0.018 | 0.999(0.964-1.035) | 0.947 |
|  | Weighted median | 22 | 0.004 | 0.010 | 1.004(0.984-1.024) | 0.705 |
|  | Weighted mode | 22 | 0.004 | 0.012 | 1.004(0.980-1.029) | 0.730 |
| **Head and neck** | MR Egger | 22 | -0.027 | 0.021 | 0.974(0.935-1.014) | 0.216 |
|  | Weighted median | 22 | -0.028 | 0.012 | 0.972(0.949-0.995) | **0.020** |
|  | Weighted mode | 22 | -0.034 | 0.018 | 0.966(0.933-1.002) | 0.075 |

**Abbreviation**: MR: Mendelian randomization; SNPs: single-nucleotide polymorphisms; SE: standard error; OR: odd ratio; confidence interval.

Bold values indicate statistical significance (*p*<0.05).
