# Supplementary material for: Assessing the role of lipid-lowering therapy on multi-cancer prevention: A mendelian randomization study
Source: Front Pharmacol. 2023 Apr 19;14:1109580. doi: 10.3389/fphar.2023.1109580 (PMC10154601; doi:10.3389/fphar.2023.1109580)
Supplement: Supplementary file 2 [file DataSheet1.ZIP › Table S5 simvastatin.docx]

**Table S5.** Two sample MR analysis results of Simvastatin use and pan-cancer.

| **Outcome** | **MR Method** | **No. SNPs** | **β** | **SE** | **OR (95%CI)** | ***P*** |
| --- | --- | --- | --- | --- | --- | --- |
| **Bladder** | MR Egger | 39 | 0.002 | 0.009 | 1.002 (0.985-1.019) | 0.838 |
|  | Weighted median | 39 | 0.001 | 0.006 | 1.001(0.989-1.013) | 0.915 |
|  | Weighted mode | 39 | 0.006 | 0.008 | 1.006(0.989-1.022) | 0.498 |
| **Lung** | MR Egger | 39 | 0.009 | 0.012 | 1.009(0.986-1.033) | 0.435 |
|  | Weighted median | 39 | -0.003 | 0.008 | 0.997(0.981-1.014) | 0.747 |
|  | Weighted mode | 39 | -0.006 | 0.011 | 0.994(0.973-1.015) | 0.559 |
| **Bile duct** | MR Egger | 34 | 0.001 | 0.007 | 1.001(0.988-1.014) | 0.900 |
|  | Weighted median | 34 | 0.002 | 0.003 | 1.002(0.996-1.009) | 0.491 |
|  | Weighted mode | 34 | 0.002 | 0.004 | 1.002(0.994-1.010) | 0.568 |
| **Liver cell** | MR Egger | 33 | -0.005 | 0.006 | 0.996(0.985-1.007) | 0.432 |
|  | Weighted median | 33 | -0.002 | 0.002 | 0.998(0.994-1.003) | 0.508 |
|  | Weighted mode | 33 | -0.004 | 0.003 | 0.996(0.991-1.002) | 0.219 |
| **Cervical** | MR Egger | 37 | -0.005 | 0.011 | 0.995(0.974-1.015) | 0.606 |
|  | Weighted median | 37 | -0.006 | 0.007 | 0.994(0.980-1.008) | 0.406 |
|  | Weighted mode | 37 | -0.007 | 0.009 | 0.993(0.975-1.011) | 0.431 |
| **Colorectal** | MR Egger | 39 | 0.019 | 0.018 | 1.019(0.984-1.056) | 0.297 |
|  | Weighted median | 39 | 0.014 | 0.012 | 1.014(0.990-1.039) | 0.259 |
|  | Weighted mode | 39 | 0.032 | 0.015 | 1.032(1.002-1.064) | 0.045 |
| **Ovarian** | MR Egger | 39 | -0.011 | 0.014 | 0.989(0.963-1.017) | 0.447 |
|  | Weighted median | 39 | 0.003 | 0.011 | 1.003(0.982-1.024) | 0.801 |
|  | Weighted mode | 39 | 0.000 | 0.013 | 1.000(0.975-1.025) | 0.977 |
| **Non-** **melanoma** | MR Egger | 39 | -0.048 | 0.047 | 0.953(0.869-1.045) | 0.314 |
|  | Weighted median | 39 | -0.014 | 0.025 | 0.986(0.940-1.035) | 0.576 |
|  | Weighted mode | 39 | -0.011 | 0.026 | 0.989(0.939-1.041) | 0.668 |
| **Melanoma** | MR Egger | 39 | -0.013 | 0.013 | 0.987(0.962-1.012) | 0.304 |
|  | Weighted median | 39 | -0.006 | 0.010 | 0.994(0.975-1.014) | 0.563 |
|  | Weighted mode | 39 | -0.008 | 0.012 | 0.992(0.969-1.015) | 0.476 |
| **Prostate** | MR Egger | 39 | -0.181 | 1.134 | 0.834(0.090-7.706) | 0.874 |
|  | Weighted median | 39 | -0.187 | 0.395 | 0.829(0.382-1.799) | 0.635 |
|  | Weighted mode | 39 | -0.390 | 0.382 | 0.677(0.320-1.432) | 0.314 |
| **Breast** | MR Egger | 39 | 0.342 | 1.123 | 1.408(0.156-12.720) | 0.762 |
|  | Weighted median | 39 | 0.171 | 0.496 | 1.187(0.449-3.138) | 0.730 |
|  | Weighted mode | 39 | -0.062 | 0.493 | 0.940(0.358-2.470) | 0.901 |
| **Oesophagus** | MR Egger | 39 | -0.005 | 0.007 | 0.995(0.982-1.008) | 0.439 |
|  | Weighted median | 39 | -0.004 | 0.004 | 0.996(0.987-1.004) | 0.315 |
|  | Weighted mode | 39 | -0.004 | 0.005 | 0.996(0.986-1.007) | 0.507 |
| **Head and neck** | MR Egger | 39 | 0.007 | 0.007 | 1.007(0.993-1.021) | 0.327 |
|  | Weighted median | 39 | 0.004 | 0.005 | 1.004(0.994-1.015) | 0.432 |
|  | Weighted mode | 39 | 0.005 | 0.007 | 1.005(0.992-1.019) | 0.460 |

**Abbreviation**: MR: Mendelian randomization; SNPs: single-nucleotide polymorphisms; SE: standard error; OR: odd ratio; confidence interval.
