# Supplementary material for: Assessing the role of lipid-lowering therapy on multi-cancer prevention: A mendelian randomization study
Source: Front Pharmacol. 2023 Apr 19;14:1109580. doi: 10.3389/fphar.2023.1109580 (PMC10154601; doi:10.3389/fphar.2023.1109580)
Supplement: Supplementary file 2 [file DataSheet1.ZIP › Table S6 Rosuvastatin.docx]

**Table S6** Two sample MR analysis results of rosuvastatin use and pan-cancer.

| **Outcome** | **MR Method** | **No. SNPs** | **β** | **SE** | **OR (95%CI)** | ***P*** |
| --- | --- | --- | --- | --- | --- | --- |
| **Bladder** | MR Egger | 6 | 0.000 | 0.320 | 1.000(0.534-1.873) | 0.999 |
|  | Weighted median | 6 | 0.046 | 0.060 | 1.047(0.931-1.179) | 0.443 |
|  | Weighted mode | 6 | 0.064 | 0.083 | 1.067(0.907-1.255) | 0.472 |
| **Lung** | MR Egger | 6 | 0.120 | 0.366 | 1.128(0.550-2.313) | 0.759 |
|  | Weighted median | 6 | -0.081 | 0.083 | 0.922(0.784-1.085) | 0.328 |
|  | Weighted mode | 6 | -0.083 | 0.114 | 0.920(0.736-1.151) | 0.499 |
| **Bile duct** | MR Egger | 6 | -0.305 | 0.147 | 0.737(0.552-0.984) | 0.107 |
|  | Weighted median | 6 | -0.064 | 0.033 | 0.938(0.878-1.001) | 0.054 |
|  | Weighted mode | 6 | -0.085 | 0.050 | 0.919(0.834-1.012) | 0.148 |
| **Liver cell** | MR Egger | 6 | -0.250 | 0.144 | 0.779(0.587-1.033) | 0.158 |
|  | Weighted median | 6 | -0.064 | 0.023 | 0.938(0.897-0.981) | **0.005** |
|  | Weighted mode | 6 | -0.075 | 0.027 | 0.927(0.879-0.979) | **0.040** |
| **Cervical** | MR Egger | 6 | 0.091 | 0.317 | 1.095(0.588-2.040) | 0.789 |
|  | Weighted median | 6 | -0.126 | 0.075 | 0.882(0.761-1.022) | 0.094 |
|  | Weighted mode | 6 | -0.126 | 0.114 | 0.882(0.706-1.102) | 0.320 |
| **Colorectal^*^** | MR Egger | 6 | 0.059 | 0.769 | 0.989(0.902-1.085) | 0.495 |
|  | Weighted median | 6 | 0.114 | 0.141 | 1.121(0.851-1.477) | 0.416 |
|  | Weighted mode | 6 | 0.103 | 0.217 | 1.108(0.725-1.695) | 0.655 |
| **Ovarian** | MR Egger | 6 | 0.747 | 0.465 | 2.110(0.849-5.245) | 0.183 |
|  | Weighted median | 6 | 0.006 | 0.122 | 1.006(0.793-1.276) | 0.963 |
|  | Weighted mode | 6 | -0.016 | 0.208 | 0.984(0.654-1.480) | 0.942 |
| **Non-** **melanoma** | MR Egger | 6 | -0.138 | 1.004 | 0.871(0.122-6.235) | 0.897 |
|  | Weighted median | 6 | -0.105 | 0.242 | 0.901(0.561-1.447) | 0.666 |
|  | Weighted mode | 6 | -0.202 | 0.315 | 0.817(0.441-1.515) | 0.550 |
| **Melanoma** | MR Egger | 6 | 0.382 | 0.433 | 1.466(0.628-3.422) | 0.427 |
|  | Weighted median | 6 | 0.018 | 0.105 | 1.018(0.829-1.250) | 0.866 |
|  | Weighted mode | 6 | 0.021 | 0.134 | 1.021(0.784-1.329) | 0.884 |
| **Oesophagus** | MR Egger | 6 | 0.236 | 0.194 | 1.266(0.865-1.853) | 0.292 |
|  | Weighted median | 6 | 0.011 | 0.048 | 1.011(0.921-1.110) | 0.813 |
|  | Weighted mode | 6 | 0.011 | 0.070 | 1.011(0.881-1.161) | 0.878 |
| **Head and neck** | MR Egger | 6 | 0.020 | 0.237 | 1.020(0.641-1.625) | 0.936 |
|  | Weighted median | 6 | -0.050 | 0.055 | 0.951(0.854-1.060) | 0.368 |
|  | Weighted mode | 6 | -0.058 | 0.082 | 0.944(0.803-1.108) | 0.511 |

**Abbreviation**: MR: Mendelian randomization; SNPs: single-nucleotide polymorphisms; SE: standard error; OR: odd ratio; CI: confidence interval.

*calculated via the MR Egger (bootstrap)

Bold values indicate statistical significance (*p*<0.05).
